# A neuron–glia lipid metabolic cycle couples daily sleep to mitochondrial homeostasis

**DOI:** 10.1038/s41593-023-01568-1

**Published:** 2024-02-15

**Authors:** Paula R. Haynes, Elana S. Pyfrom, Yongjun Li, Carly Stein, Vishnu Anand Cuddapah, Jack A. Jacobs, Zhifeng Yue, Amita Sehgal

**Affiliations:** 1 Department of Neuroscience, University of Pennsylvania, Philadelphia, PA USA; 2 Chronobiology and Sleep Institute, Perelman School of Medicine at the University of Pennsylvania, Philadelphia, PA USA; 3 Howard Hughes Medical Institute, University of Pennsylvania, Philadelphia, PA USA; 4 Children’s Hospital of Philadelphia, University of Pennsylvania, Philadelphia, PA USA

**Keywords:** Sleep, Glial biology

## Abstract

Sleep is thought to be restorative to brain energy homeostasis, but it is not clear how this is achieved. We show here that *Drosophila* glia exhibit a daily cycle of glial mitochondrial oxidation and lipid accumulation that is dependent on prior wake and requires the *Drosophila* APOE orthologs NLaz and GLaz, which mediate neuron–glia lipid transfer. In turn, a full night of sleep is required for glial lipid clearance, mitochondrial oxidative recovery and maximal neuronal mitophagy. Knockdown of neuronal *NLaz* causes oxidative stress to accumulate in neurons, and the neuronal mitochondrial integrity protein, Drp1, is required for daily glial lipid accumulation. These data suggest that neurons avoid accumulation of oxidative mitochondrial damage during wake by using mitophagy and passing damage to glia in the form of lipids. We propose that a mitochondrial lipid metabolic cycle between neurons and glia reflects a fundamental function of sleep relevant for brain energy homeostasis.

## Abstract (web summary)

Haynes et al. report a daily, sleep-dependent neuron–glia lipid metabolic cycle. ApoE-dependent lipid transfer from neurons to glia protects neurons from oxidative damage during waking, and lipids are cleared from glia during sleep.

## Main

The function of sleep remains a major mystery in biology, with little known about why prolonged wakefulness generates the drive to sleep. It is likely that energy consumption during wake contributes to sleep need, but the nature of this energetic imbalance and how it is resolved by sleep is not known. In addition to conserving energy, sleep is thought to promote the clearance of toxic proteins and metabolites via different pathways^1–3^.

Glia are increasingly implicated in the regulation of sleep^4–6^, largely with respect to their role in neuromodulation. However, glia also have important roles in brain energetics, which contribute to pathology when disrupted. For instance, Liu et al. proposed that unchecked neuronal mitochondrial damage results in the accumulation and subsequent lysis of toxic lipid droplets in glia, leading to neurodegeneration^7^. Others have also shown that glial lipid droplets accumulate as a result of high levels of neuronal activity^8^. It is possible that, even under healthy conditions, lipid transport from neurons to glia provides a mechanism for neurons to transfer products of energetic stress to glia for breakdown and/or detoxification.

As mitochondria are critical mediators of neuronal energy production, we considered the possibility that mitochondrial-based signaling is key in linking brain energy metabolism to daily sleep. Here, we report a sleep-regulated metabolic cycle across neurons and glia that allows for the integration of mitochondrial and lipid metabolism into a brain energy homeostasis-based model of daily sleep.

## Results

### Wakefulness causes oxidation of glial mitochondria

Although the generation of sleep need on a cellular basis has been described for a specific circuit in *Drosophila*^9,10^, it is clear that sleep is a phenomenon of the whole brain. Indeed, the need for sleep can even arise locally in non-sleep circuits subjected to wake-associated activity (local sleep)^11,12^. As waking activity is energetically demanding, and neurons in particular require large amounts of mitochondrial energy to repolarize after firing, we asked if wake increases the oxidation of neuronal or glial mitochondria in a brain-wide fashion.

**Fig. 1:**
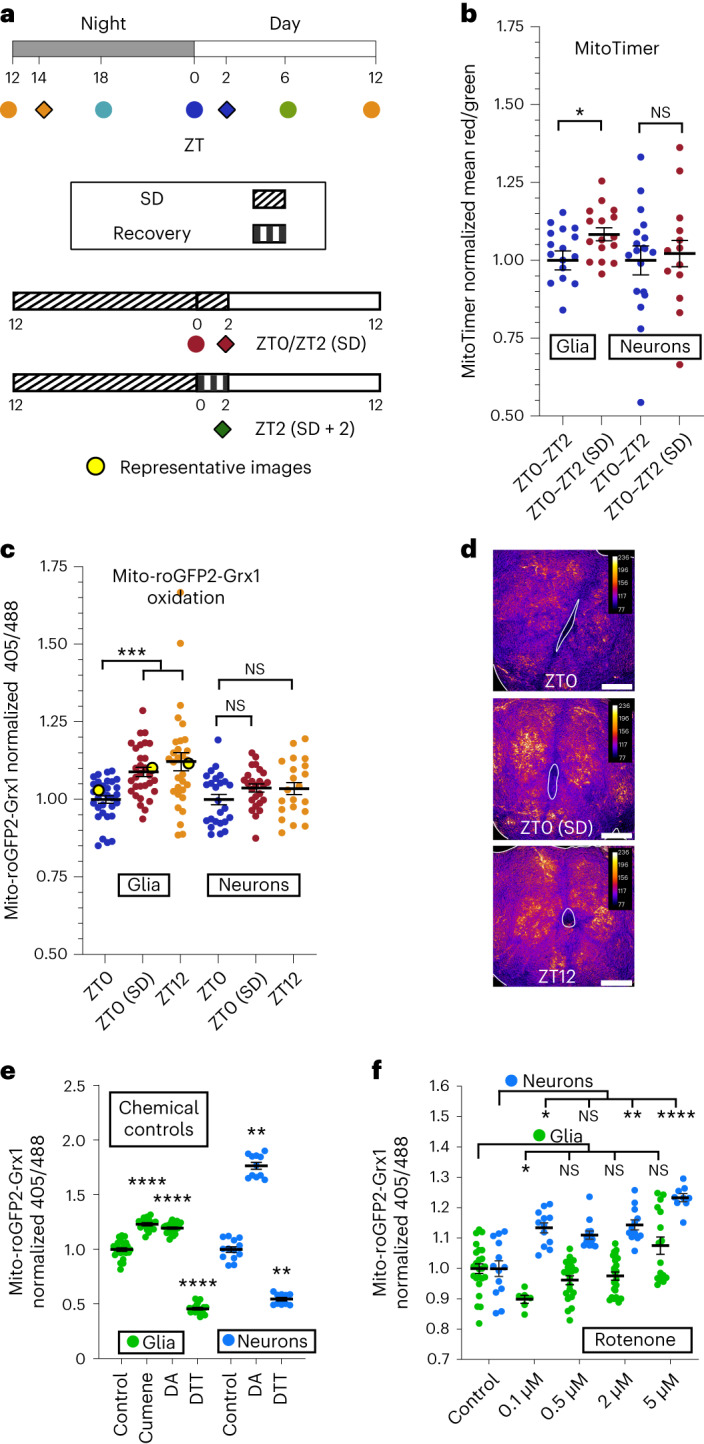
Wakefulness promotes oxidation of glial mitochondria. **a**, Schematic showing light/dark entrainment, sleep deprivation (SD) and fly collection times for all experiments. Top, flies were collected at the indicated Zeitgeber Times, ZT. Bottom, Flies were sleep deprived for 12–14 h before collection at ZT0/2. If sleep recovery in the first 2 h of the morning was allowed, collection times for all groups were shifted by 2 h (ZT2/ZT14, filled diamonds/squares). **b**, Neuron/glia MitoTimer. Left, glial mitochondrial oxidation is increased in sleep-deprived flies compared to in control flies with a full night of sleep. Right, neuronal mitochondrial oxidation is not significantly affected by sleep deprivation; *P* = 0.73; NS, not significant. **c**–**f**, Neuron/glia mito-roGFP2-Grx1. Glial mitochondrial oxidation is increased in flies at the end of the wake period (ZT12) and in sleep-deprived flies (ZT0; **c**, left). Neuronal mitochondrial oxidation is not significantly affected at the end of the wake period (ZT12) or by sleep deprivation; *P* ≥ 0.24 (**c**, right). Representative brains from glial mito-roGFP2-Grx1 experiments show the central brain with antennal lobes central and facing up (**d**; false colored with the fire LUT). Data points from the representative images are highlighted in yellow in **c**; scale bars, 50 μm each. **e**, Brains expressing mito-roGFP-Grx1 in neurons or glia were exposed to the chemical oxidizing agent DA (5 mM), the chemical reducing agent DTT (5 mM) or the lipid peroxide modeling compound cumene hydroperoxide (cumene; 2 mM). **f**, Brains were incubated with increasing doses of the complex I inhibitor rotenone (0.1, 0.5, 2 and 5 μM). Rotenone at all doses induced increases in neuronal, but not glial, mito-roGFP2-Grx1 oxidation. At 0.1 μM rotenone, glia exhibited a decrease in oxidation. In **b**–**f**, *repo*-GAL4 was used for glial expression, and *nSyb*-GAL4 was used for neuronal expression. For all data shown, *P < 0.05, **P < 0.01, ***P < 0.001 and ****P < 0.0001. Bars/error bars indicate mean and s.e.m., respectively. Data points indicate individual flies/brains. The following are the numbers of flies (*n*) as plotted from left to right and the statistical tests used: glia *n* = 17 and 16 and neurons *n* = 16 and 16, Mann–Whitney test (two tailed; **b**); *n* = 32, 31, 31, 25, 21 and 25, Kruskall–Wallis test with a Dunn’s multiple testing correction (**c**); glia *n* = 24, 16, 22 and 18, analysis of variance (ANOVA), Fisher’s least significant different test, uncorrected; neurons *n* = 13, 11 and 10, Kruskall–Wallis test with a Dunn’s post hoc test, uncorrected (**e**); glia *n* = 24, 6, 21, 22 and 16, Kruskall–Wallis test with a Dunn’s multiple testing correction; neurons *n* = 13, 12, 12, 12 and 9, Kruskall–Wallis test with a Dunn’s multiple testing correction (**f**). Source data

Using the mitochondrially targeted fluorescent protein MitoTimer, which converts from green to red fluorescence excitation/emission when oxidized^13^, we found, surprisingly, that although flies deprived of sleep for one night showed increases in glial MitoTimer oxidation, no changes were apparent in neuronal MitoTimer oxidation (Fig. 1a,b). MitoTimer is able to detect increases in neuronal mitochondrial oxidation in *Drosophila*^10^, suggesting that sensor saturation in neurons does not explain our observation.

**Extended Data Fig. 1:**
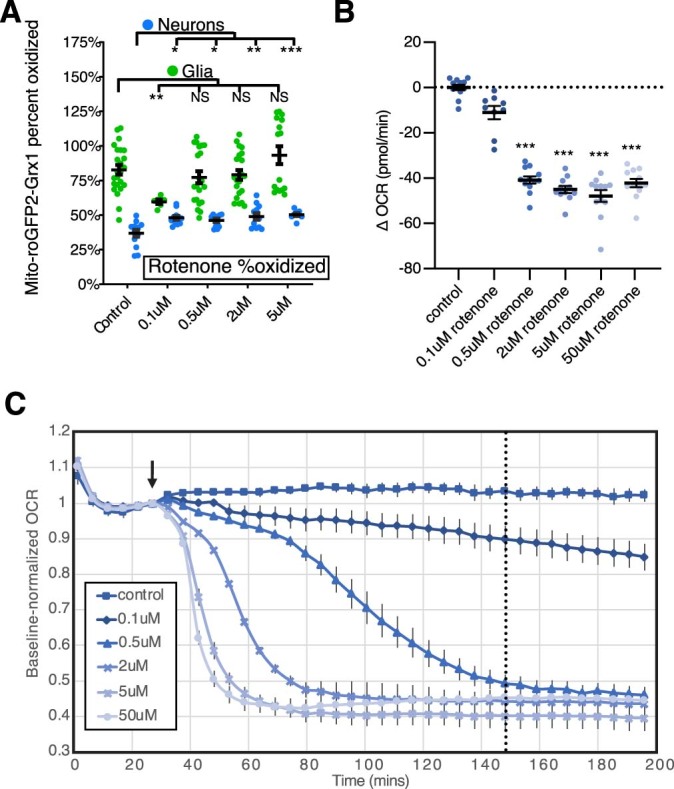
Controls for mito-roGFP-Grx1 sensor function show that ROS-induced inhibition of mitochondrial CI respiration results in significant oxidation of neuronal mitochondria. **(A)** The data in main Fig. 1e, f was used to calculate percent oxidation of rotenone-exposed neurons and glia relative to the specific minimum (DTT) and maximum (DA) oxidation levels in each cell type. The data shown here is the same as that in Fig. 1f, but presented in terms of percent oxidized for each cell type (calculation as indicated in Methods). **(B)** A Seahorse XFe96 system was used to measure oxygen consumption of individual *Drosophila* brains in the presence of increasing concentrations of the mitochondrial complex-I inhibitor, rotenone. OCR is significantly reduced following a 2 hr exposure to 0.5uM or greater doses of rotenone. ΔOCR was calculated for each brain as the change in OCR after 120 mins of rotenone exposure minus the baseline OCR at 27 mins. ΔOCR values were pooled within each group. **(C)** The Oxygen Consumption Rate (OCR in pmol/min) of each brain over time is shown normalized to its baseline OCR after a 27-minute stabilization period, indicated by the arrow, just prior to rotenone injection. The dotted line at 147 mins (120 mins after rotenone injection) indicates the timepoint used for quantification of ΔOCR values in B. For all data shown, *=p < 0.05, **=p < 0.01 and ***=p < 0.001 while some groups with p > 0.05 (not significant) are unmarked. Bars/ error bars indicate mean and SEM, respectively. Data points indicate individual brains. Number of flies/brains (n) as plotted from left to right and statistical test used: (A) n = 24,6,21,22,16; all Kruskall-Wallis-Dunn’s MC (B-C) n = 12,9,12,11,12,12; Kruskall-Wallis-Dunn’s MC. MC = corrected for multiple comparisons. Source data

To verify and extend our MitoTimer findings, we used an additional mitochondrial redox sensor specific to the glutathione reducing pool, mito-roGFP2-Grx1 (ref. ^14^). After oxidation-induced disulfide bond formation, mito-roGFP2-Grx1 converts from 488 nm to UV excitation and effectively reports increases in mitochondrial^15^ or cytosolic^16^ oxidation in *Drosophila* neurons. Confirming our MitoTimer results with mito-roGFP2-Grx1, we again found that glial, but not neuronal, mitochondria are significantly more oxidized following a night of sleep deprivation (Zeitgeber time 0 (ZT0; sleep deprivation)) and also following a day of wake (ZT12; Fig. 1c,d). The capacity of neuronal and glial mito-roGFP2-Grx1 to respond to increases and decreases in mitochondrial oxidation status was confirmed by incubating brains in the chemical oxidizing or reducing agents diamide (DA) or dithiothreitol (DTT), respectively (Fig. 1e). Neurons exhibited a larger dynamic range between minimum (DTT) and maximum (DA) oxidation fluorescence than glia (neuronal DA/DTT ratio = 3.26 and glial DA/DTT ratio = 2.61; similar to a previous report^16^), and neuronal controls were less oxidized (36%) than glial controls (81%; Fig. 1e and Extended Data Fig. 1a). To confirm the ability of mito-roGFP2-Grx1 to detect physiologically relevant increases in mitochondrial oxidation in neurons as well as differences between neurons and glia, brains were incubated for 2 h in rotenone, a mitochondrial complex I inhibitor, which induces oxygen radical formation. Acute rotenone treatment caused significant increases in neuronal mito-roGFP2-Grx1 signal and no increases in glial mito-roGFP2-Grx1 signal (Fig. 1f). The high sensitivity of neurons, but not glia, to acute oxidative stress with rotenone treatment is consistent with the known redox buffering capacity of these respective cell types^17,18^.

Use of a Seahorse XFe96 system to analyze changes in respiration (oxygen consumption rate (OCR)) showed that although neuronal mito-roGFP2-Grx1 is sensitive to complex I inhibition with 0.1 μM rotenone, 0.1 μM rotenone does not significantly impair respiration within the same time frame (Extended Data Fig. 1b,c). These control experiments, together with the experiments in Fig. 1e,f, demonstrate that neuronal mito-roGFP2-Grx1 is highly sensitive to changes in neuronal mitochondrial oxidation status, particularly under acute stress conditions as with rotenone, but not with wake or sleep deprivation. Our findings indicate that wake-dependent mitochondrial oxidation is primarily a glial phenomenon in healthy animals.

Accumulation of wake-dependent mitochondrial oxidation in glia, but not neurons, is unexpected given the known metabolic specializations of neurons and glia. Whereas glia are primarily glycolytic and contain a lower density of mitochondria, neurons are more energetically demanding, contain a high density of mitochondria and rely primarily on mitochondria for energetic substrates^17^. Thus, neurons are expected to produce greater levels of mitochondrial oxidation, yet we observe accumulation of mitochondrial oxidation only in glia. This raises the possibility that oxidation-inducing substrates, which accumulate during wake, are transferred from neurons to glia.

A number of studies document damage-induced transfer of proteins and lipids from neurons to glia^7,19^, even including the transfer of whole mitochondria in some cases^20^. Additionally, a subset of antioxidant genes with little to no expression in neurons is highly expressed by glia, suggesting that glia may be better equipped than neurons to detoxify certain classes of oxidized proteins and lipids^17,18^. In fact, following neuronal mitochondrial damage, loss of neuron-to-glia lipid transfer results in neurodegeneration^7^. Thus, lipid transport may provide a mechanism for neurons to transfer oxidizing equivalents to glia for detoxification. To determine if peroxidated lipids could be responsible for wake-driven oxidation of glial mito-ro-GFP2-Grx1, brains were incubated in the lipid peroxide inducing and modeling compound cumene hydroperoxide (2 mM)^21,22^, which resulted in maximal oxidation of the sensor (Fig. 1e). This indicates that glial mito-ro-GFP2-Grx1 is sensitive either directly or indirectly (via reduced-glutathione depletion) to oxidation by lipid peroxides.

### Wakefulness causes glial lipid droplet accumulation

**Fig. 2:**
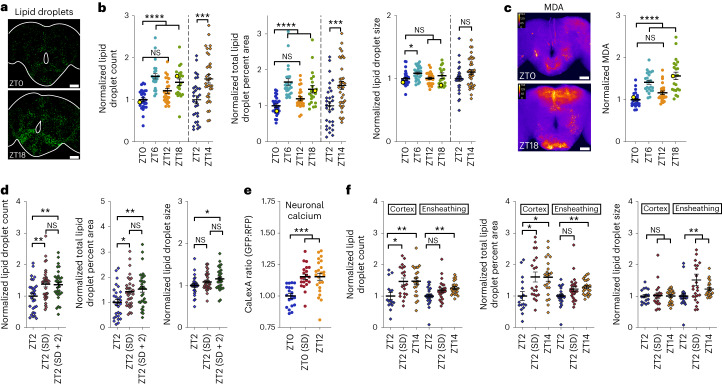
Lipid droplets accumulate in glia following wake and neuronal activity. **a**,**b**, Images of representative brains from lipid droplet experiments (**a**; data points in yellow in **b**) with BODIPY 493 brain lipid droplet staining. Lipid droplet count (**b**, left), percentage of brain area occupied (**b**, middle) and lipid droplet size (**b**, right) are shown; scale bars, 50 μm. **c**, Antibody staining for MDA. Right, central brain MDA. Left, images of representative brains from MDA staining experiments (data points in yellow on right); scale bars, 50 μm. **d**, Flies were sleep deprived for 14 h (ZT2 SD) or 12 h with 2 h of recovery sleep (ZT2 SD + 2). Lipid droplet count (left), percentage of brain area occupied (middle) and lipid droplet size (right) were quantified. **e**, Pan-neuronal (*nSyb*-GAL4) CaLexA-GFP shows that calcium is broadly increased in the brain following normal wake (ZT12) or a night of sleep deprivation (ZT0 SD); RFP, red fluorescent protein. **f**, A day of wake (ZT14) or a night (14 h) of sleep deprivation (ZT2 + SD) induces the accumulation of lipid droplets in the cortex and ensheathing glial subsets as quantified by UAS-*Lsd-2*-GFP. Increased lipid droplet count (left) in cortex glia (NP2222-GAL4) and ensheathing glia (MZ0709-GAL4) are apparent at ZT14 and ZT2 + SD. The percentages of brain area occupied by lipid droplets (middle) and lipid droplet size (right) in glial subsets are shown. Color and protocol designations are the same as in Fig. 1a. In **b** and **d**, ZT0/ZT6/ZT12/ZT18 (white Canton-S flies) were a separate set of experiments from ZT2/ ZT14 (Iso31). See Methods for further information. For all data shown, *P < 0.05, **P < 0.01, ***P < 0.001 and ****P < 0.0001, while some groups with *P* > 0.05 (not significant) are unmarked. Bars/error bars indicate mean and s.e.m., respectively. Data points indicate individual flies/brains. The following are the numbers of flies (*n*) as plotted from left to right and statistical tests used: ZT0/ZT6/ZT12/ZT18 *n* = 25, 24, 24 and 21, Kruskall–Wallis test with a Dunn’s multiple testing correction; ZT2/ZT14 *n* = 29 and 34, unpaired two-tailed *t*-test (**b**); *n* = 24, 22, 24 and 21, Kruskall–Wallis test with a Dunn’s multiple testing correction (**c**); *n* = 29, 33 and 33, ANOVA with a Holm–Sidak multiple testing correction (**d**); *n* = 20, 21 and 23, Kruskall–Wallis test with a Dunn’s multiple testing correction (**e**); *n*_Cortex_ = 16, 20 and 22; *n*_Ensheathing_ = 24, 24 and 23; cortex, left and center: ANOVA with a Holm–Sidak multiple testing correction; cortex, right, and all ensheathing: Kruskall–Wallis test with a Dunn’s multiple testing correction (**f**). Source data

**Extended Data Fig. 2:**
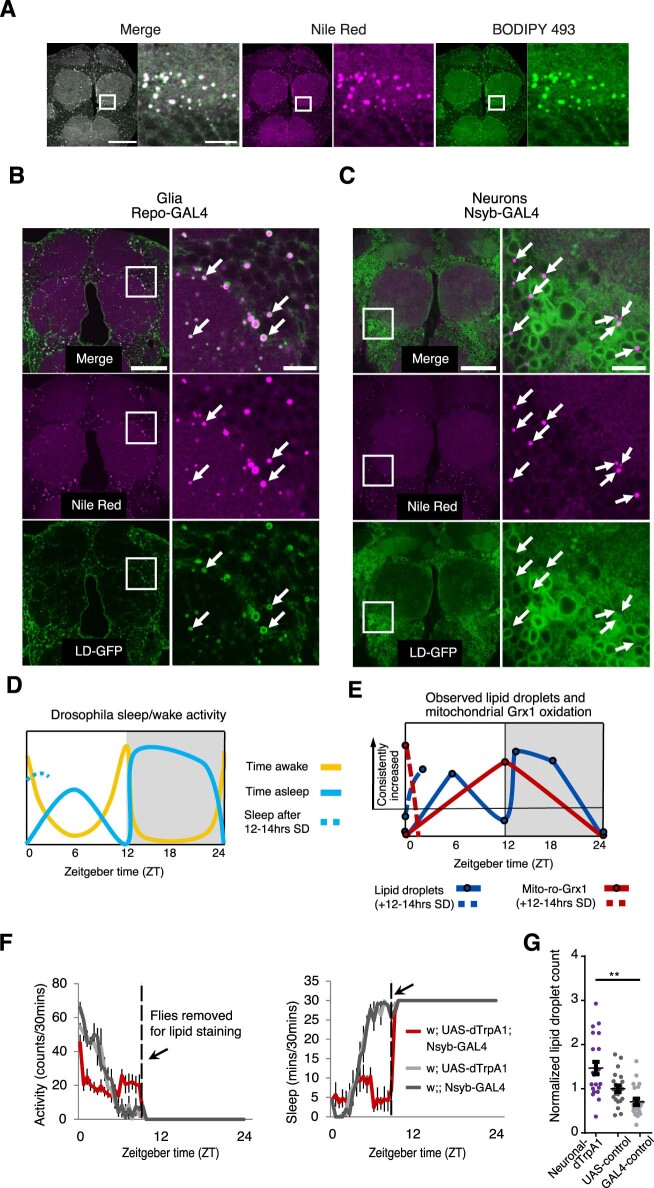
Central brain lipid droplets in *Drosophila* are localized primarily to glia and increase with neural activity. **(A)** The lipophilic dyes, BODIPY 493 and Nile Red, colocalize in *Drosophila* brains and can be used interchangeably. Here, the anterior region of a single, central brain is shown stained with both dyes. (A, right- green) BODIPY 493 staining, (A, center -magenta) Nile Red staining and (A, left) merge of both channels with colocalization in white. (n = 6 brains were imaged to verify colocalization between BODIPY and Nile Red.) **(B and C)** The lipid droplet marker, UAS-LD-GFP, was expressed in glia with Repo-GAL4 or in neurons with Nsyb-GAL4 and stained with the lipophilic dye, Nile Red. **(B)** LD-GFP expressed in glia was found to localize in a ring around nearly all Nile-Red stained lipid droplets (n = 7/7 of brains imaged showed GFP and Nile Red colocalization). **(C)** Conversely, neuronally-expressed LD-GFP exhibited a very bright, diffuse localization pattern in neuropil and the periphery of cell bodies and was *never* seen in a ring pattern around *any* lipid droplets stained by Nile Red (n = 0/7 of brains imaged showed GFP and Nile Red colocalization). For all images, the central brain is shown on the left with a white box indicating the enlarged region shown on the right. Arrows mark the locations of representative lipid droplets. Scale bars at left are 50uM each and at right (enlarged) are 10uM each. All images were acquired with sequential, independent imaging of red and green excitation/emission. Proper dye-staining protocols can be found in the Methods section and were critical for visualization of central brain lipid droplets. **(D)** Known patterns of time spent awake (yellow) and time spent asleep (blue) throughout the day in *Drosophila* on a 12:12 light: dark cycle or following sleep deprivation (dotted). **(E)** Schematic summarizing experimental timepoints from all figures, connected by lines, from lipid droplet count (dark blue) and glial mito-roGFP2-Grx1 oxidation (red) experiments showing when each metric is or is not consistently increased. While both dependent on prior wake, increases in mito-roGFP2-Grx1 oxidation are most consistent during known wake periods (ZT0 + SD and ZT12, as in D), while lipid droplet count only increases consistently during periods with some sleep (ZT2 + SD, ZT6, ZT14, ZT18, as in D). In flies on RU-486, mito-roGFP-Grx1 is most consistently increased after SD and lipid droplets are most consistently increased at ZT18. **(F)** Activity counts per 30 minutes (left) and sleep per 30 minutes (right) of flies with dTrpA1-induced neuronal hyperactivation (red) and controls (grey). The temperature was increased from 23 °C to 25.5 °C at ZT0 and remained at 25.5°C from ZT0-ZT9. **(G)** Hyperactivation of all neurons with Nsyb>dTrpA1 (25.5 °C) from ZT0-ZT9 causes a further increase in lipid droplet accumulation over controls. For all data shown, *=p < 0.05, **=p < 0.01, ***=p < 0.001 and ****=p < 0.0001. Bars/ error bars indicate mean and SEM, respectively. Data points indicate individual flies/brains. Number of flies (n) as plotted from left to right and statistical test used: (F-G) n = 22,23,23; Mann-Whitney-two tailed. Source data

If oxidizing equivalents in neurons increase with wake and are transferred to glia as lipids, they should result in wake-dependent lipid droplet accumulation in glia. To test this idea, we used the neutral lipid stain BODIPY 493 to quantify central brain lipid droplet accumulation following periods of sleep or wake. Because glia primarily, and neurons only rarely, produce lipid droplets in mammals in vivo^8,23,24^ and in *Drosophila*^25–28^, the vast majority of observed staining should represent glial lipid droplets. We confirmed this with the lipid droplet marker LD–green fluorescent protein (LD–GFP)^29^, which formed rings around the majority of brain lipid droplets when expressed in glia and showed a complete absence of lipid droplet localization when expressed in neurons (Extended Data Fig. 2a–c). LD–GFP is a fusion of GFP with the lipid droplet-targeting domain of Klar-β, which localizes to existing lipid droplets but does not induce droplet formation on its own^29^. With BODIPY 493 staining for lipid droplets, we found that lipid droplets are increased during sleep periods following wake (ZT6, ZT14 and ZT18) but not at peak wake times (ZT0, ZT2 and ZT12; Fig. 2a,b and Extended Data Fig. 2d,e). To determine if the lipid droplets accumulating after wake are associated with peroxidated lipids, we used an antibody to the lipid peroxidation breakdown product malondialdehyde (MDA). We found that MDA is increased after wake at the same time points as lipid droplets (ZT6 and ZT18; Fig. 2c). Importantly, we also found that lipid droplet accumulation is driven by homeostatic sleep need, increasing after a night of sleep deprivation (Fig. 2d). Together, these findings indicate that lipid droplet accumulation during sleep is associated with peroxidated lipids, is driven by homeostatic sleep need and occurs as a normal physiological function of the sleep–wake cycle. Additionally, because sleep need driven by prior time awake is high at ZT12, but we do not observe a consistent increase in lipid droplets until later (ZT14–ZT18; Fig. 2b and Extended Data Fig. 2d,e), additional factors such as sleep depth or the circadian clock likely gate sleep need-driven lipid droplet accumulation during sleep periods.

Wake is associated with increased levels of neuronal activity^30^, which may be responsible for the wake-dependent lipid droplet accumulation that we observe. To verify that wake promotes general increases in neuronal activity, we expressed the calcium indicator CaLexA^31^ in all neurons and collected flies at the end of wake (ZT12) or following a night of sleep deprivation (ZT0 SD). We observed that this is a general brain-wide phenomenon (Fig. 2e). Thus, the wake-induced lipid droplet accumulation and oxidative stress that we observe may be, at least in part, a function of increased neuronal activity during wake. Indeed, pathologically increased levels of neuronal activity were shown to increase glial lipid droplets in mammalian systems^8^, so we sought to determine if this could also occur in *Drosophila*. To increase waking neuronal activity broadly across the brain, we expressed the temperature-sensitive cation channel dTrpA1 in all neurons and modestly activated neurons at 25.5 °C for 9 h during the wake period. This manipulation reduced total sleep and altered the distribution of locomotor activity over time, although total activity counts were not significantly different from controls (Extended Data Fig. 2f). Following 9 h of neuronal hyperactivation, we observed an increase in glial lipid droplets relative to unperturbed controls at the same time point (ZT9; Extended Data Fig. 2g). Although this is clearly a non-physiological manipulation not directly comparable to normal wake, it shows that increased neuronal activity is capable of promoting lipid droplet accumulation, and, together with our CaLexA results, it suggests that the increased neuronal activity that occurs during wake may promote the accumulation of glial lipid droplets.

**Extended Data Fig. 3:**
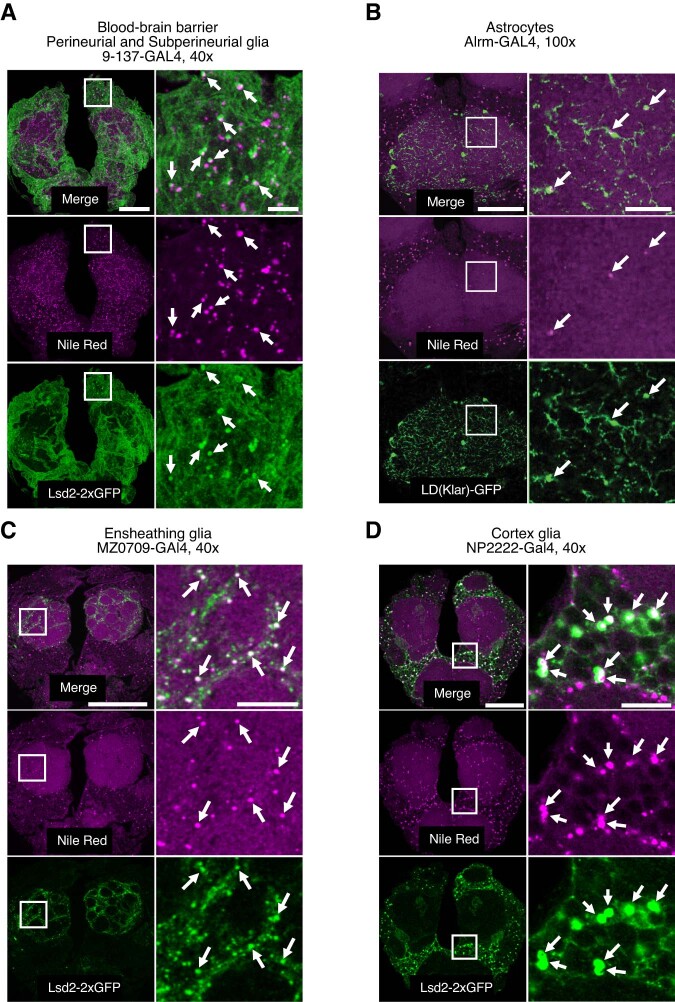
Lipid droplets in the *Drosophila* brain are localized primarily to cortex and ensheathing glia. Glial subset-specific GAL4 lines were used to express the LD-GFP or Lsd2-GFP, and brains were stained with the lipophilic dye, Nile Red. Colocalization of subset-specific LD/Lsd2-GFP puncta with Nile-red stained lipid droplets was taken as indication of the capacity for lipid droplet formation within a given glial subset. **(A)** Blood-brain barrier (BBB) glia encompassing the perineurial and subperineurial populations (9-137-GAL4), contain a small number of lipid droplets. Generally, no more than ~20 lipid droplets per brain could be observed in the BBB and counts did not appear to be obviously altered by a night of sleep deprivation. (n = 9 non-deprived and n = 9 sleep-deprived brains). In order to illustrate the presence of BBB lipid droplets, the images shown are a maximum projection of 3-confocal Z-planes showing an unusually dense region of BBB lipid droplet expression. **(B)** Astrocytes expressing LD-GFP in the Alrm-GAL4 expression pattern form only a few, sparse lipid droplets (n = 3 brains). The region shown is of the subesophageal area of the central brain (100x magnification). Other neuropil areas exhibited a similar sparseness of astrocytic lipid droplets. **(C)** Ensheathing glia (MZ0709-GAL4) contain large numbers of very small lipid droplets. The majority of Nile-Red stained lipid droplets within neuropil regions appear to localize to neuropil compartment boundaries where ensheathing glia are located, as shown here surrounding antennal lobe glomeruli. (n = 10/10 brains imaged). **(D)** Cortex glia (NP2222-GAL4) contain a high density of larger lipid droplets. Nearly all Nile-Red-stained lipid droplets outside of neuropil regions localize to cortex glia. (n = 8/8 brains imaged). For all images, the central brain is shown on the left with a white box indicating the enlarged region shown on the right. Scale bars at left are 50uM each and at right (enlarged) are 10uM each. The bottom panel of each group of images shows Lsd2/LD-GFP expression driven by the respective GAL4, the central panel shows Nile Red staining of the same brain/z-slice and the top panel shows a merge of the green and red channels. Arrows mark the locations of representative lipid droplets. All images were acquired with sequential imaging of red and green excitation/emission to prevent any bleed-through between channels. Source data

The *Drosophila* brain contains a number of functionally distinct glial subpopulations^32^. Cortex glia, which surround neuronal cell body compartments, have been characterized as containing large numbers of lipid droplets in larvae^25^. To identify the glial subsets that accumulate lipid droplets in adults, glial subset-specific GAL4 lines^33^ were used to drive expression of the tagged lipid droplet coat protein UAS-*Lsd-2*-GFP (refs. ^34,35^) or LD–GFP^29^. We confirmed the presence of lipid droplets in cortex glia and found that ensheathing glia also contain large numbers of lipid droplets, whereas blood–brain barrier glia and astrocytes contain very few lipid droplets (Extended Data Fig. 3). Because cortex and ensheathing glia contained the vast majority of central brain lipid droplets, we used pan-glial Lsd-2–GFP to quantify wake-dependent lipid droplet accumulation in these populations. As with pan-glial, dye-based experiments (Fig. 2a,b,d), we found that both cortex and ensheathing glia accumulate lipid droplets in a wake-dependent manner (Fig. 2f). Lipid droplet count and relative percentage of brain area occupied by droplets were increased in both populations of glia following a day of wake (ZT14) and following sleep deprivation, although this increase only reached significance in cortex glia (Fig. 2f, left and middle). Ensheathing glia droplets instead showed a significant increase in lipid droplet size with sleep deprivation (Fig. 2f, right). These results demonstrate that glia that compartmentalize neuronal cell body and neuropil regions (cortex and ensheathing glia, respectively) have specialized roles in wake-dependent lipid droplet accumulation in *Drosophila*.

### GLaz and NLaz are necessary for glial mitochondrial oxidation

**Fig. 3:**
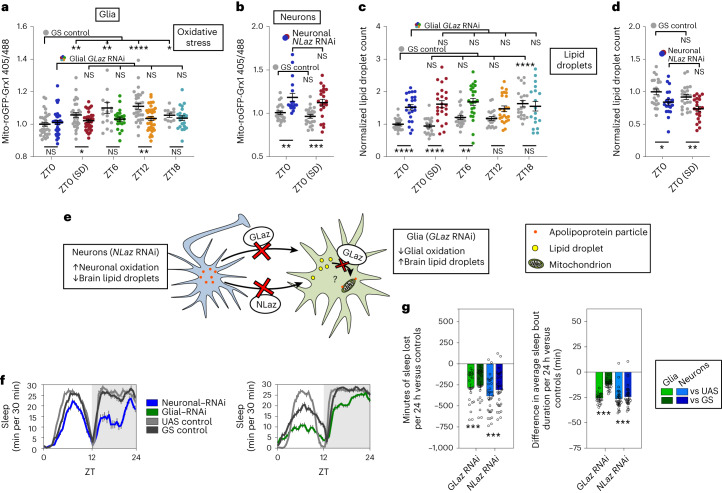
Adult-specific knockdown of lipid transport genes in neurons or glia causes sleep loss, alters cell-type-specific mitochondrial oxidation and impairs glial lipid droplet processing. **a**–**d**, Oxidative stress (mito-roGFP-Grx1; **a** and **b**) and lipid droplets (**c** and **d**) in glia (left) and neurons (right). The experimental GS>RNAi genotypes are shown in time point-specific colors (as in Fig. 1a), whereas the GS control genotypes are in gray. **e**, Schematic illustrating the effects of neuronal *NLaz* RNAi or glial *GLaz* RNAi on mito-roGFP2-Grx1 oxidation in neurons and glia, respectively, or on brain lipid droplets. Increased lipid droplets with glial *GLaz* RNAi suggests that GLaz may play an additional role in the delivery of lipids to mitochondria for breakdown. **f**, Total sleep (30-min bins) is reduced with adult-specific knockdown of *NLaz* in neurons (left) or *GLaz* in glia (right). Gray shading indicates the dark period. **g**, Adult-specific knockdown of the lipid transport genes *GLaz* in glia (green) or *NLaz* in neurons (blue) results in sleep loss (left) and reduced sleep bout duration (right). Sleep loss is represented as sleep of the experimental genotype minus the average sleep of each of the control groups (UAS or GS). *repo*-GS; Dcr with/without *GLaz* RNAi (V15389) and *nSyb*-GS; Dcr with/without *NLaz* RNAi (V35558) were used for all glial and neuronal experiments, respectively, in this figure along with mito-roGFP-Grx1 in non-sleep experiments. For **a**–**d**, statistical differences across time points and within genotype are shown above the plotted points, whereas differences between experimental and control genotypes at each time point are shown below. For all data, bars/error bars indicate mean and s.e.m., respectively, where error due to subtraction between groups in sleep data has been propagated in the s.e.m. bars shown. Data points indicate individual flies/brains. For all data shown, *P < 0.05, **P < 0.01, ***P < 0.001 and ****P < 0.0001, while some groups with *P* > 0.05 (not significant) are unmarked. The following are the numbers of flies (*n*) as plotted from left to right and statistical tests used: *n* = 33, 30, 33, 33, 21, 21, 33, 33, 14 and 21; ZT0/ZT6/ZT12/ZT18, Kruskall–Wallis test with a Dunn’s multiple testing correction; ZT0/ZT0 SD (all comparisons), Mann–Whitney test (two tailed) except GS versus GS RNAi, which was analyzed by an unpaired two-tailed *t*-test (**a**); *n* = 22, 18, 22 and 21 (all comparisons), Mann–Whitney test (two tailed) except ZT0/ZT0 SD (GS versus GS), which was analyzed by an unpaired *t*-test (two tailed; **b**); *n* = 21, 23, 20, 24, 22, 24, 20, 24, 21 and 18; ZT0/ZT6/ZT12/ZT18, Kruskall–Wallis test with a Dunn’s multiple testing correction; ZT0/ZT0 SD (all comparisons), unpaired two-tailed *t*-test except ZT0/ZT0 SD (GS versus GS RNAi), which was analyzed by a two-tailed Mann–Whitney test (**c**); *n* = 21, 20, 22 and 21, all unpaired two-tailed *t*-test (**d**); *n* = 25, 25, 31 and 31, all Mann–Whitney test (two tailed; **f** and **g**). Source data

We have shown that lipid droplets accumulate in glia during sleep in a fashion dependent on prior wake, but it is not clear if this accumulation requires lipid transfer from neurons to glia, if it plays any role in wake-dependent oxidation of glial mitochondria or if it protects against oxidation of neuronal mitochondria. The glial and neuronal fatty acid transport proteins GLaz and NLaz, respectively, are functional orthologs of mammalian apolipoprotein E (ApoE) and are both independently required for the transfer of lipids from neurons to glia^36^. If neuron-to-glia lipid transfer is necessary for the wake-dependent increase in glial mitochondrial oxidation that we report, then inhibiting the expression of *GLaz* should reduce glial mitochondrial oxidation. As expected, the control group showed increased glial mitochondrial oxidation at all time points compared to ZT0, but in the presence of *GLaz* RNA interference (RNAi), differences between time points were abolished (Fig. 3a, within-genotype statistics at the top). In particular, knocking down *GLaz* expression in glia reduced mito-roGFP2-Grx1 oxidation levels at the end of the wake period, at ZT12 and following sleep deprivation (Fig. 3a, between-genotype statistics at the bottom).

If neuron-to-glia lipid transfer serves to protect neurons from oxidative damage, we would also expect to see that inhibiting lipid transfer with neuronal *NLaz* RNAi would increase neuronal mitochondrial oxidation. In fact, this is exactly what we see at ZT0 both with and without sleep deprivation, indicating that lipid transfer is necessary to protect neuronal mitochondria and that sleep (ZT0, non-deprived) is unable to ameliorate neuronal oxidative damage when lipids are not transferred to glia (Fig. 3b). This indicates that glial lipid uptake via GLaz and NLaz is directly responsible for both the increased oxidation of glial mitochondria with sleep deprivation and the lack of neuronal oxidation under the same conditions (Fig. 1c).

If the wake-driven glial lipid droplets that we observe in Fig. 2 are dependent on neuron–glial lipid transfer, we would also expect knockdown of glial *GLaz* or neuronal *NLaz* to reduce lipid droplets following wake. Surprisingly, we found that knockdown of glial *GLaz* increases lipid droplets in the first half of the day (ZT0 with and without SD and ZT6; Fig. 3c, bottom comparisons), whereas neuronal *NLaz* RNAi results in the expected decrease in lipid droplets (Fig. 3d). The decreased lipid droplets and increased neuronal mitochondrial oxidation with neuronal *NLaz* RNAi is consistent with an impairment of wake-driven neuron–glia lipid and oxidative damage transfer (Fig. 3b). Likewise, glial *GLaz* RNAi protects glial mitochondria from wake-dependent increases in oxidation, although the increased lipid droplets with glial *GLaz* RNAi in the first half of the day were not predicted by loss of lipid transfer. Instead, this observation suggests an additional function of GLaz in lipid breakdown during sleep (Fig. 3e). The results of the experiments in Fig. 3a–d thus indicate wake-driven NLaz- and GLaz-mediated lipid and oxidative damage transfer from neurons to glia as well as possible GLaz-mediated lipid catabolism (summarized in Fig. 3e).

**Extended Data Fig. 4:**
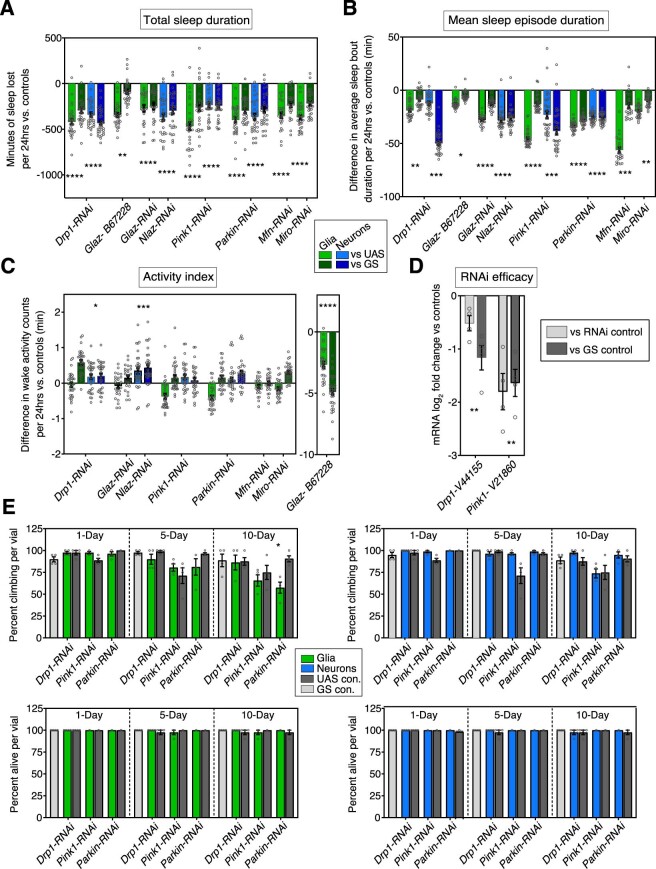
RNAi knockdown of lipid transfer or mitochondrial damage control genes: additional sleep parameters, qPCR, negative geotaxis and survival. Knockdown of lipid transfer and mitochondrial damage control genes reduces total sleep duration **(A)**, and mean sleep episode duration **(B)** without general effects on activity index **(C)**. RNAi was adult-induced with Nsyb-GS;Dcr or Repo-GS;Dcr: *Drp1*-RNAi (V44155), *Glaz*-RNAi (V15389) *Nlaz*-RNAi (V35558) *Pink1*-RNAi (V21860), *Parkin*-RNAI (V47636), Mfn-RNAi (B67158), Miro-RNAi (V330334). Neuronal, but not glial, *Drp1*-RNAi expression results in an increased activity index when flies are awake and does not occur with an alternative *Drp1*-RNAi insertion or dominant negative *Drp1*(Extended Data Fig. 5). Neuronal *Nlaz*-RNAi, but neither glial *Glaz*-RNAi construct results in an increased activity index, suggesting increased activity index is not general phenomenon associated with lipid-transfer RNAis. An alternative *Glaz*-RNAi line (B67228) exhibited sleep loss and fragmentation **(A-B)** in spite of a severe activity index deficit **(C, right)**. **(D)** *Drp1*-(V44155) or *Pink1*-(V21860) RNAi expression was induced with Actin5C-GS for 5-6 days and the difference in mRNA expression between experimental and UAS/GS controls was quantified by qPCR. *Nlaz*, *Glaz*, *Pink1*, *Parkin*, *Mfn* and *Miro*-RNAi knockdown have been verified elsewhere, as indicated in the Methods section. **(E)** Negative geotaxis (top) and survival (bottom) with adult-induced expression of *Drp1*-RNAi (V44155), *Pink1*-RNAi (V21860) or *Parkin*-RNAi (V47636) in neurons (blue) or glia (green). Neuronal knockdown does not affect negative geotaxis or survival within the first 10-days while glial knockdown of *Pink1* or *Parkin* causes negative geotaxis at 10-days only. Glial *Drp1*-RNAi animals are unaffected. Bars/error bars indicate mean and SEM, respectively, where error due to subtraction between groups in sleep data has been propagated in the SEM bars shown. For sleep data, individual points indicate sleep of individual experimental flies minus the average sleep of the respective control group. P-values shown are the greater (least significant) of these two sets of comparisons, where *=p < 0.05, **=p < 0.01, ***=p < 0.001 and ****=p < 0.0001. Number of flies (n) as plotted from left to right and statistical test used: (A-C) n = 31,31,32,32,32,32,25,25,31,31,30,30,31,31,32,32,32,31,31,25,25,32,32; all Mann-Whitney-2T (D) n = 4 biological replicates with 10 flies per genotype in each replicate; paired T-test-2T (E) n = 4 vials (replicates) per genotype with 10 flies per vial; unpaired T-test-2T. 2T = two-tailed. Source data

To address the behavioral relevance of neuron-to-glia lipid transfer, we asked if blocking this transfer affects daily sleep. Thus, we induced knockdown of *GLaz* in glia or *NLaz* in neurons of adult flies and found a reduction in and fragmentation of sleep (Fig. 3f,g). Neuronal *NLaz* RNAi also resulted in an increased activity index, suggesting that hyperactivity may contribute to the neuronal phenotype; however, this is not the case for glial *GLaz* RNAi (Extended Data Fig. 4c). This finding and our *GLaz* and *NLaz* RNAi mito-roGFP2-Grx1 and lipid droplet results mechanistically link wake-dependent neuron–glia lipid transfer to sleep, showing that brain-wide lipid metabolic interactions between neurons and glia are necessary for normal daily sleep and reflect a homeostatic lipid redox function of sleep.

### *Drp1* knockdown disrupts daily lipid droplet cycles

Our results in Fig. 3 indicate that wake-dependent lipid transfer to glia reflects a mechanism to curb mitochondrial damage accumulation in neurons. If so, then mitochondrial damage-response proteins may be required for one or both of these processes. One such protein is Drp1, a dynamin-like GTPase that mediates mitochondrial fission and selective mitophagy. Drp1 activity is promoted by reactive oxygen species (ROS)-mediated *S*-nitrosylation^37,38^ and increases with oxidized glutathione^15,39^. Interestingly, Drp1 is also activated through dephosphorylation by the sleep-promoting phosphatase calcineurin^40,41^ and is inhibited by phosphorylation at the same site by the wake-promoting kinase, protein kinase A^42,43^.

**Fig. 4:**
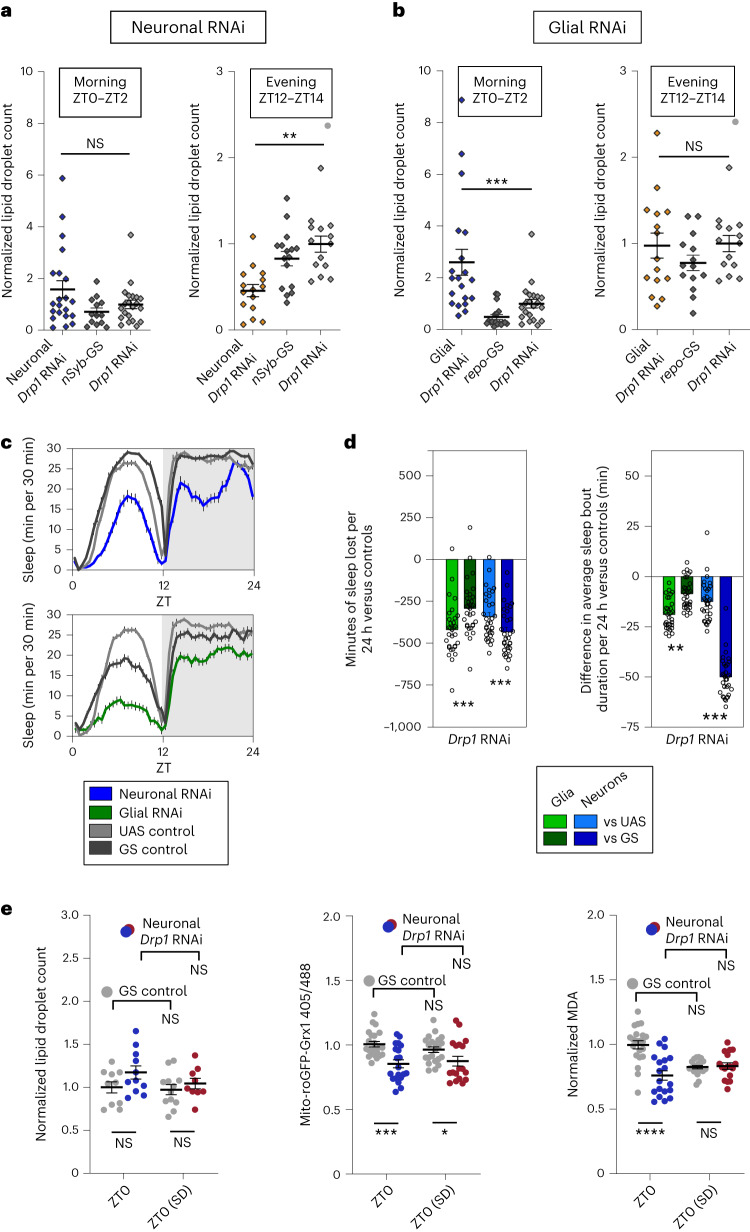
Accumulation of lipid droplets during wake depends on Drp1 in neurons while their clearance during sleep requires glial Drp1. **a**,**b**,**e**, Lipid droplets were stained with BODIPY 493. **a**, Knockdown of *Drp1* in neurons does not affect brain lipid droplet levels following sleep (left, ZT0–ZT2) but causes decreased lipid droplet accumulation after wake (right, ZT12–ZT14). **b**, Knockdown of *Drp1* in glia causes an increase in brain lipid droplets following sleep (left, ZT0–ZT2) but does not affect lipid droplet accumulation after wake (right, ZT12–ZT14). **c**,**d**, Adult-specific knockdown of *Drp1* in neurons (blue) or in glia (green) reduces total sleep (30-min bins (**c**) and 24-h bins (**d**, left)) and sleep bout duration (**d**, right). **e**, Neuronal *Drp1* RNAi does not alter brain lipid droplet count (left) but reduces mito-roGFP2-Grx1 oxidation at ZT0 (middle) and brain MDA (right). Flies for lipid droplet experiments in **a** and **b** were collected from ZT0 to ZT2 or from ZT12 to ZT14, whereas flies in **e** were collected at ZT0. In **e**, statistical differences across time points and within genotype are shown above the plotted points, whereas differences between experimental and control genotypes at each time point are shown below. *repo*-GS; Dcr or *nSyb*-GS; Dcr with/without *Drp1* RNAi (V44155) were used for all glial and neuronal experiments, respectively. In **d**, sleep loss is represented as sleep of the experimental genotype minus the average sleep of each of the control groups (UAS or GS). Data points indicate individual flies/brains. For all data, bars/error bars indicate mean and s.e.m., respectively, where error due to subtraction between groups in sleep data has been propagated in the s.e.m. bars shown. For all data shown, *P < 0.05, **P < 0.01, ***P < 0.001 and ****P < 0.0001, while some groups with *P* > 0.05 (not significant) are unmarked. The following are the numbers of flies (*n*) as plotted from left to right and statistical tests used: *n* = 21, 13, 21, 15, 16 and 14, all comparisons Mann–Whitney test (two tailed; **a**); *n* = 20, 18, 21, 15, 14, 14 and 14, all comparisons Mann–Whitney test (two tailed; **b**); *n* = 31, 31, 32 and 32, all comparisons Mann–Whitney test (two tailed; **c** and **d**); *n* = 10, 11, 12 and 9, all comparisons unpaired two-tailed *t*-test (**e**, left); *n* = 21, 17, 21 and 19, all comparisons Mann–Whitney test (two tailed) except GS versus GS, which was analyzed by unpaired two-tailed *t*-test (**e**, middle); *n* = 21, 19, 21 and 17, all comparisons unpaired two-tailed *t*-test (**e**, right). Source data

To determine if neuronal Drp1 activity is required for wake-driven glial lipid accumulation, we knocked down *Drp1* in neurons and quantified central brain lipid droplet levels. Knockdown of *Drp1* in neurons resulted in a reduction of lipid droplets during sleep and at the end of the wake period (ZT12–ZT14) when control lipid droplet levels are greatest, but not in the morning (ZT0–ZT2) when control lipid droplet levels are low (Fig. 4a). This supports the idea that the transfer of lipids from neurons to glia requires mitochondrial damage control mechanisms in neurons.

**Fig. 5:**
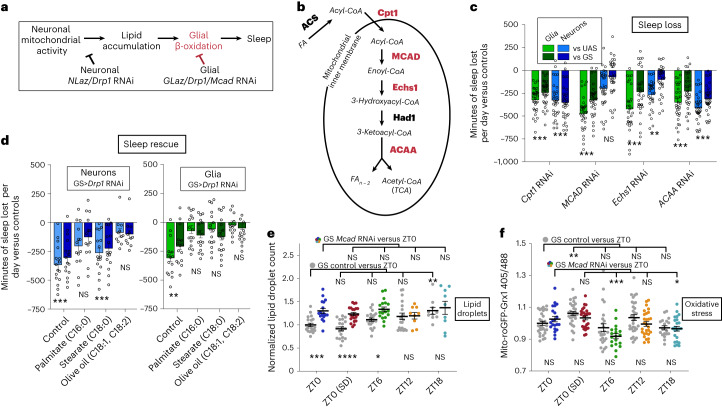
β-Oxidation of fatty acids in glia is required for sleep. **a**, Model for lipid transporter and *Drp1*-knockdown effects on brain lipid droplets and sleep. If glial mitochondrial β-oxidation (red) is required for breakdown of lipids and sleep, glial *Mcad* or *Drp1* RNAi should result in increased lipid accumulation and reduced sleep. **b**, *Drosophila* genes involved in mitochondrial fatty acid (FA) β-oxidation. Genes driving enzymatic steps are shown in bold, whereas fatty acid modifications are italicized. Red indicates the genes tested and shown to reduce sleep with RNAi expression in glia (RNAis were not tested for genes in black); TCA, tricarboxylic acid cycle. **c**, Adult-induced knockdown of fatty acid catabolism genes. *Mcad* and *Echs1* in glia (green) cause greater sleep loss per 24 h than neuronal knockdown (blue). **d**, Dietary fatty acid supplementation rescues sleep loss resulting from neuronal (left, blue) or glial (right, green) *Drp1* RNAi (V44155). The comparisons shown are between the experimental (GS>RNAi) and control genotypes (GS or RNAi) on each FA; within-genotype comparisons are shown in Extended Data Fig. 7a. **e**,**f**, Adult-induced glial *Mcad* RNAi disrupts lipid droplet processing during sleep (**e**) and the dynamics of glial mitochondrial oxidative stress as measured with mito-roGFP2-Grx1 (**f**). For **c** and **d**, individual points indicate sleep of individual experimental flies minus the average sleep of the respective control group. *repo*-GS; Dcr and *nSyb*-GS; Dcr with/without RNAis were used for all glial and neuronal experiments, respectively. In **c** and **d**, sleep loss is represented as sleep of the experimental genotype minus the average sleep of each of the control groups (UAS or GS). Data points indicate individual flies/brains. For all data, bars/error bars indicate mean and s.e.m., respectively, where error due to subtraction between groups in sleep data has been propagated in the s.e.m. bars shown. For all data shown, *P < 0.05, **P < 0.01, ***P < 0.001 and ****P < 0.0001, while some groups with *P* > 0.05 (not significant) are unmarked. The following are the numbers of flies (*n*) as plotted from left to right and statistical tests used: *n* = 32, 32, 32, 32, 32, 32, 31, 31, 30, 30, 31, 31, 32, 32, 32, 32 and 32, all comparisons Mann–Whitney test (two tailed; **c**); neurons *n* = 16, 16, 14, 14, 16, 16, 11 and 11 and glia = 15, 15, 16, 16, 16, 16, 14 and 14, all comparisons Mann–Whitney test (two tailed; **d**); *n* = 20, 17, 20, 21, 21, 18, 19, 9, 10 and 9, ZT0/ZT6/ZT12/ZT18, all ANOVA with a Holm–Sidak multiple testing correction except GS versus GS, which was analyzed by a Kruskall–Wallis test with a Dunn’s multiple testing correction; ZT0/ZT0 SD, all unpaired two-tailed *t*-test (**e**); *n* = 31, 24, 30, 26, 28, 21, 32, 28, 21 and 23, ZT0/ZT6/ZT12/ZT18, all Kruskall–Wallis test with a Dunn’s multiple testing correction; ZT0/ZT0 SD, all Mann–Whitney test (two tailed; **f**). Source data

In glia, mitochondrial activity is required to drive lipid catabolism^26,27^. If *Drp1* is necessary for glial mitochondrial lipid catabolism, knockdown of *Drp1* in glia should result in increased lipid droplet levels at a time when they have normally been catabolized. In fact, this is exactly what we see; central brain lipid droplet levels were higher in the morning (ZT0–ZT2), when control levels were low, and were no different from controls at the end of the wake period (ZT12–ZT14), when control levels were highest (Fig. 4b). This supports the idea that glial mitochondrial activity is responsible for lipid droplet breakdown during sleep (see model in Fig. 5a). The experiments in Fig. 5 further support a role for glial mitochondrial activity in lipid breakdown, specifically through β-oxidation.

### Mitochondrial damage control genes are required for normal sleep

Thus far, we have shown that Drp1 activity in neurons is required for glial lipid droplet accumulation during wake, whereas Drp1 in glia is required for clearance of lipid droplets during sleep. Although these findings indicate a function for daily sleep, it is not clear if this function of sleep is mechanistically linked to the generation of normal sleep itself. To determine if Drp1 is required for sleep, we used *nSyb*-GS and *repo*-GS to drive adult-inducible RNAi in either neurons or glia, respectively. We found that adult-induced knockdown of *Drp1* in either neurons or glia results in sleep loss and fragmentation, the latter manifesting as reduced duration of sleep episodes (Fig. 4c,d). We also observed that knockdown of the additional mitochondrial damage control genes *Pink1*, *park*, *Marf* and *Miro* in glia and/or neurons also results in sleep loss and fragmentation (Extended Data Fig. 4a–c). Although constitutive loss of expression of the mitochondrial stress-response genes *Pink1*, *park* and *Mul1* has been linked to circadian rhythms and sleep–wake timing in *Drosophila*^44–47^, our results provide evidence for a role of mitochondrial stress-response genes in daily sleep quality and quantity.

**Extended Data Fig. 5:**
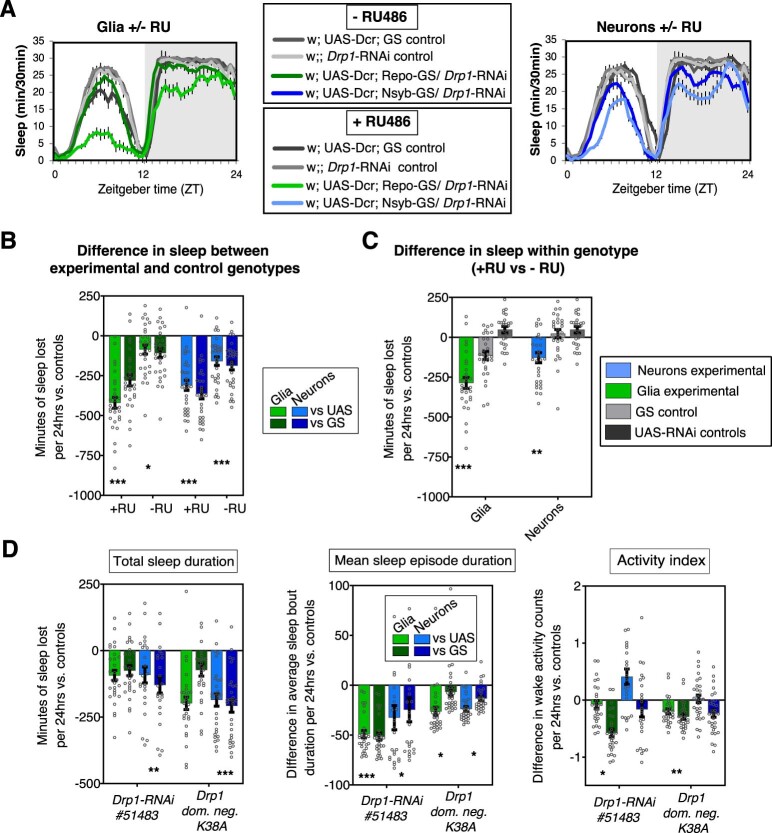
*Drp1*-knockdown sleep phenotypes are driven by RU486-specific induction and corroborated by alternative methods of *Drp1* knockdown. **(A-C)** Repo-GS;Dcr or Nsyb-GS;Dcr was used to drive *Drp1*-RNAi (V44155) expression in glia or neurons, respectively, and total sleep was compared for all genotypes in the presence or absence of the GS activator, RU-486. **(A)** Total sleep per 30 minutes of glial (left, green) and neuronal (right, blue) experimental and control groups with or without RU486 in the food. **(B)** Difference in total sleep between the experimental and control genotypes on the same food (+RU or –RU). The experimental genotypes show some sleep loss both in the presence and absence of RU-486, as has been reported previously, however sleep loss is significantly greater in the presence of RU-486, indicating additional adult-specific induction. **(C)** Difference in total sleep as compared within each genotype in the presence or absence of RU-486. In spite of some leakiness, experimental groups (Repo/Nsyb-GS>*Drp1*-RNAi) show greater sleep loss in the presence of RU486 than in the absence of RU486, while control groups show no consistent differences. **(D)** Repo-GS;Dcr or Nsyb-GS;Dcr was used to drive an alternative RNAi against *Drp1* (B51483), as well as over-expression of dominant-negative *Drp1* (K38A) in glia or neurons, respectively. **(D, left)** Total sleep duration is reduced with neuronal knockdown of *Drp1* expression/activity. **(D, center)** Mean sleep episode duration is reduced with knockdown of *Drp1* expression/activity in neurons or glia. **(D, right)** activity index is reduced with knockdown of *Drp1* expression/activity in glia. Bars/error bars indicate mean and SEM, respectively, where error due to subtraction between groups has been propagated in the SEM bars shown. Individual points indicate sleep of individual experimental flies minus the average sleep of the respective control group. P-values shown are the greater (least significant) of these two sets of comparisons, where *=p < 0.05, **=p > 0.01 and ***=p < 0.001. Groups with p > 0.05 (not significant) are unmarked. Number of flies (n) as plotted from left to right and statistical test used: (A-B) n = 29,29,30,30,30,30,30,30; all comparisons - Mann-Whitney-2T (C) n = 29,30,28,30,30,28; all comparisons - Mann-Whitney-2T (D, all) n = 28,28,23,23,29,29,29,29; all comparisons - Mann-Whitney-2T. 2T = two-tailed. Source data

To verify the efficacy and specificity of the *Drp1* RNAi effects, we performed additional experiments. The knockdown efficacy of *Drp1* RNAi was confirmed by quantitative PCR (qPCR; Extended Data Fig. 4d and Table 1). Adult-specific inducibility of sleep phenotypes was verified by comparing *Drp1* RNAi phenotypes in the presence and absence of the GeneSwitch activator RU-486 (Extended Data Fig. 5a–c). The specificity of sleep phenotypes was confirmed using an alternative RNAi line or with overexpression of dominant-negative^48^ *Drp1*^K38A^ (Extended Data Fig. 5d). Adult-induced knockdown of neuronal or glial *Drp1* for 5 or fewer days consistently reduced sleep episode duration, an indicator of sleep quality, in all lines tested. Total sleep duration was also consistently reduced with all neuronal *Drp1* manipulations tested (Extended Data Fig. 5d), and locomotor activity did not explain the reduced sleep phenotype (Extended Data Figs. 4c and 5d). We further verified that adult-induced glial or neuronal knockdown of *Drp1* within and beyond this time frame does not grossly affect the health of animals as evidenced by climbing ability and survival, which is similar in control and experimental animals at 1–10 d after RU-486 induction (Extended Data Fig. 4e).

**Extended Data Fig. 6:**
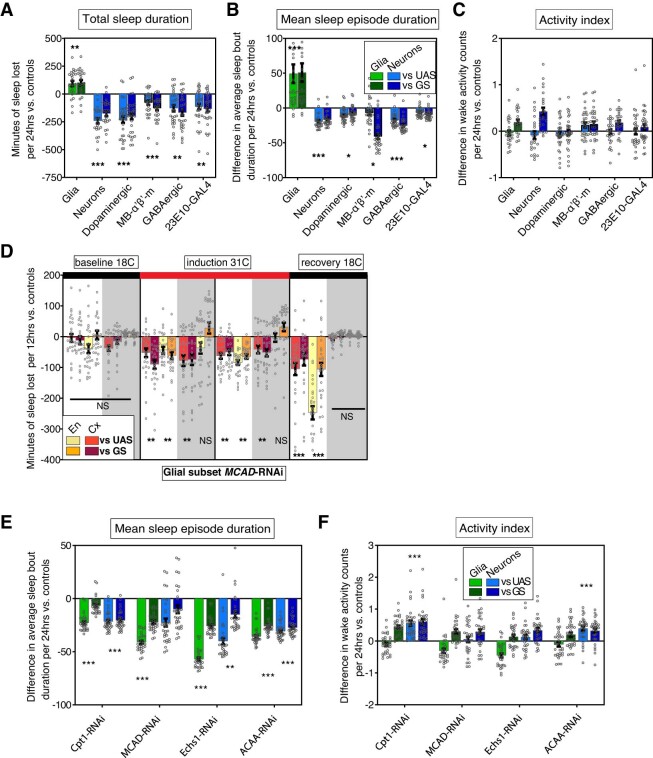
Effects of mitochondrial and beta oxidation gene expression knockdown on sleep. **(A and B)** Constitutive knockdown of *Drp1* in wake-promoting neuronal subsets (Dopaminergic/*ple*-GAL4, MB-α’β’-m/R26E01-GAL4) or sleep-promoting neuronal subsets (GABAergic/Gad2B-GAL4, dFSB/R23E10-GAL4) reduces and fragments 24 hr sleep (A and B, blue). As opposed to adult-induced induction with Geneswitch, constitutive expression of Drp1-RNAi in glia (Repo-GAL4) increases 24 hr sleep (A, green) and sleep consolidation (B, green). However, constitutive expression of Drp1-RNAi in all neurons (Nsyb-GAL4, blue) is consistent with the adult-induced sleep loss resulting from induction with Geneswitch. **(C)** Activity while awake is not significantly altered with constitutive *Drp1*-RNAi in any of these populations. The *Drp1*-RNAi line used in A-C is the same as that in Fig. 4 (VDRC #44155). **(D)** Temperature-sensitive-GAL80-mediated, adult-induced knockdown of fatty acid catabolism gene, *MCAD*, in ensheathing glia (driven by MZ0709-GAL4, yellow/orange) or cortex glia (driven by NP2222-GAL4, red) results in sleep loss. Black bars at the top indicate baseline and recovery periods at 18 °C, while the red bar indicates induction of RNAi expression (derepression of GAL4 activity) at 31 °C. Unshaded regions indicate daytime sleep change (ZT0-12), while shaded regions indicate nighttime sleep change (ZT12-24). **(E)** Mean Sleep bout duration and **(F)** activity index associated with the experiment in Fig. 5c. All glial-RNAis cause reduced mean sleep episode duration. Neuronal RNAi against *CPT1* and *ACAA* reduced sleep episode duration while increasing activity index. Bars/error bars indicate mean and SEM, respectively, where error due to subtraction between groups has been propagated in the SEM bars shown. Individual points indicate sleep of individual experimental flies minus the average sleep of the respective control group. P-values shown are the greater (least significant) of these two sets of comparisons, where *=p < 0.05, **=p < 0.01, ***=p<0.001 and ****=p < 0.0001. Groups with p > 0.05 (not significant) are unmarked. Number of flies (n) as plotted from left to right and statistical test used: (A-C) n = 23,23,24,24,31,31,28,28,28,28,32,32; all comparisons- Mann-Whitney-2T (D) n = 27 for all genotypes; all comparisons- Mann-Whitney-2T (E-F) n = 32,32,32,32,32,32,31,31,30,30,31,31,32,32,32,32,32 all comparisons- Mann-Whitney-2T. 2 T = two-tailed. Source data

Because optimal mitochondrial health is likely required for high levels of neuronal activity, we wondered if sleep loss resulting from knockdown of neuronal *Drp1* resulted from impaired activity of sleep-promoting neurons. However, we found that reductions in *Drp1* expression in both sleep- and wake-promoting neurons result in sleep loss and fragmentation (Extended Data Fig. 6a–c), indicating that neuronal mitochondrial integrity regulates sleep broadly across the brain.

Comparing adult-induced versus constitutive knockdown of *Drp1* in neurons and glia, we found that although constitutive knockdown in neurons (using *nSyb*-GAL4) resulted in sleep loss, constitutive glial knockdown (using *repo*-GAL4) suppressed sleep loss and even promoted increases in sleep (Extended Data Fig. 6a,b). This difference is likely the result of developmental compensation, highlighting the importance of differentiating between adult-induced and developmental phenotypes.

To verify that neuronal knockdown of *Drp1* does not lead to lipid droplet and sleep loss phenotypes as a result of neuronal oxidative damage, we compared lipid droplets, mitochondrial oxidation status and MDA at ZT0 with and without sleep deprivation (Fig. 4e). As we previously observed (Fig. 4a), lipid droplet levels at ZT0 are not significantly increased with neuronal *Drp1* RNAi (Fig. 4e, left), even following sleep deprivation. More importantly, and also surprisingly, we found that mitochondrial oxidation (Fig. 4e, center) and MDA (Fig. 4e, right) are not increased at baseline (ZT0) with neuronal *Drp1* RNAi and are instead decreased below baseline levels. This reduced oxidation was even maintained following sleep deprivation, whereas MDA following sleep deprivation was indistinguishable from control levels. These results verify that the mechanism by which impairment of neuron–glia lipid transfer leads to lipid droplet and sleep loss phenotypes with neuronal *Drp1* RNAi cannot be explained by increased neuronal oxidative damage.

### Mitochondrial β-oxidation in glia is required for sleep

Our findings thus far suggest that glial mitochondria are required to process lipids that accumulate into droplets following wake. This is consistent with findings demonstrating that the expression of lipid processing genes is high in glia and low or absent in neurons in both flies and mammals^49,50^. In fact, decreased lipid catabolism in glia could account for the reduced sleep produced by *Drp1* knockdown in neurons or glia despite the opposite effects of these knockdowns on lipid droplets. Neuronal *Drp1*-knockdown results in a lack of lipid substrates for catabolism, whereas glial *Drp1* knockdown likely impairs glial mitochondrial β-oxidation, resulting in an accumulation of lipids (Fig. 5a). This would be consistent with the recently discovered role for *Drp1* in promoting β-oxidation^51^.

To determine if mitochondrial β-oxidation of glial lipids is important for sleep, we acutely knocked down lipid catabolism genes in neurons and glia. *Drosophila* genes involved in mitochondrial β-oxidation are shown in Fig. 5b. We found that glial knockdown of genes involved in mitochondrial β-oxidation results in reduced and fragmented sleep (Fig. 5c and Extended Data Fig. 6e,f). Importantly, glial knockdown of key β-oxidation genes *Mcad* and *Echs1* caused much greater sleep loss than neuronal knockdown. This is in agreement with our model that glial mitochondria, specifically, are required for fatty acid processing to drive sleep.

Interestingly, knockdown of the fatty acid processing genes *whd* (*CPT1*) and *yip2* (*ACAA*) in neurons or glia resulted in similar levels of sleep loss (Fig. 5c). Because waking activity was also increased in lines with neuronal knockdown (Extended Data Fig. 6f), we were unable to distinguish changes in total sleep from hyperactivity, although sleep is probably reduced in these flies. ACAA catalyzes the final step of β-oxidation but is also involved in ketolysis, which may be indicative of a role for neuronal ketolysis in sleep. Although the localization of CPT1 in adult *Drosophila* neurons is unknown, neuronal CPT1 does not localize to mitochondria in mammalian neurons and thus may have a β-oxidation-independent function in neurons^52^.

We have shown that cortex and ensheathing glia contain the majority of lipid droplets in the brain and accumulate lipid droplets in a wake-dependent manner. To determine if β-oxidation in these particular glial subsets is important for sleep, we used temperature-sensitive GAL80 together with subset-specific GAL4s to drive *Mcad* RNAi in an adult-inducible manner (Extended Data Fig. 6d). We observed no difference in sleep between experimental and control groups at the permissive temperature (baseline, 18 °C), but at the restrictive temperature (induction, 31 °C), daytime sleep loss resulted from *Mcad* RNAi in ensheathing glia and both day and nighttime sleep loss resulted from knockdown in cortex glia. This demonstrates that normal daily sleep requires β-oxidation by glial subsets that accumulate lipid droplets in a wake-dependent manner.

**Extended Data Fig. 7:**
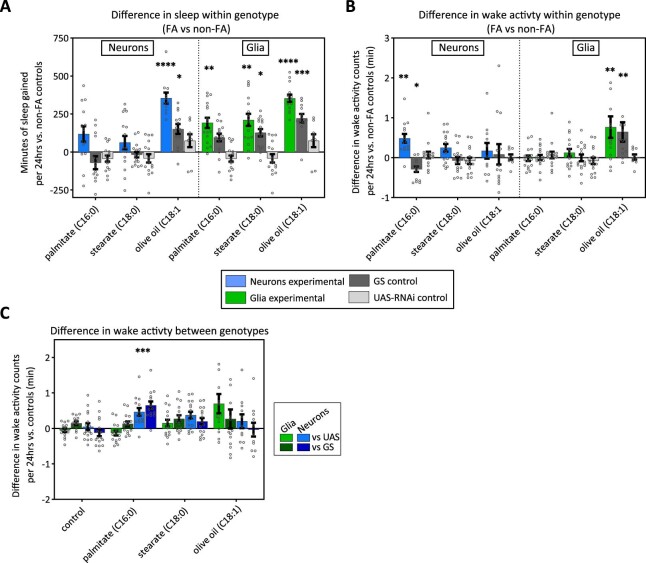
Additional comparisons and sleep parameters for *Drp1*-RNAi lines with fatty acid rescue. Additional comparisons from the experiment shown in main Fig. 5d with adult-induced neuronal (Nsyb-GS;Dcr) or glial (Repo-GS;Dcr) knockdown of *Drp1* (V44155) in the presence of fatty acid feeding. **(A)** Difference in total sleep duration within each genotype on control food or food supplemented with the indicated fatty acid. As with comparisons between genotypes (main Fig. 5d), comparisons within each genotype indicate that sleep loss with neuronal *Drp1*-RNAi is rescued only by olive oil, while sleep loss with glial knockdown is rescued by palmitate, stearate and olive oil. The Nsyb and Repo-GS control groups also show some sleep gain with fatty acid supplementation, but this gain is not as great as within the experimental groups and is not observed consistently in repeat experiments or in UAS controls. **(B)** Difference in activity index within each genotype and between genotypes **(C)** indicates fatty acid feeding does not impair locomotor activity during wake and may increase it in some conditions. Bars/error bars indicate mean and SEM, respectively, where error due to subtraction between groups has been propagated in the SEM bars shown. Individual points indicate sleep of individual experimental flies minus the average sleep of the respective control group. P-values shown are the greater (least significant) of these two sets of comparisons, where *=p < 0.05, **=p < 0.01, ***=p < 0.001 and ****=p < 0.0001. Groups with p > 0.05 (not significant) are unmarked. Number of flies (n) as plotted from left to right and statistical test used: (A-B) neurons n = 14,14,16,16,16,16,11,13,9 glia n = 16,16,16,16,16,16,14,13,9 all comparisons- Mann-Whitney-2T (C) n = 15,15,16,16,16,16,14,14,16,16,16,16,14,14,11,11 all comparisons- Mann-Whitney-2T. 2 T = two-tailed. Source data

We speculated that dietary fatty acids would substitute for the reduced lipid production resulting from neuronal *Drp1* RNAi. In fact, we found that feeding flies long-chain unsaturated fatty acids results in rescue of sleep with neuronal *Drp1* RNAi (olive oil: C18:1, C18:2), whereas long-chain saturated fatty acids (palmitate C16:0 or stearate C18:0) do not rescue sleep loss (Fig. 5d, left, and Extended Data Fig. 7a). Sleep loss with glial knockdown of *Drp1* was rescued with both types of fatty acids, suggesting that these fatty acids can be processed to affect sleep even in the absence of glial *Drp1* (Fig. 5d, right, and Extended Data Fig. 7a). Although sleep could be rescued, locomotor activity counts during wake were not generally affected by fatty acid feeding (Extended Data Fig. 7b,c). Rescue by fatty acids supports our model that lipid metabolism is important for sleep and reductions in lipid accumulation and catabolism are responsible for the sleep loss observed with neuronal or glial knockdown of *Drp1*. Although fatty acid supplementation rescues sleep loss resulting from *Drp1* RNAi, it does not consistently increase total sleep in control flies (Extended Data Fig. 7a), indicating that fatty acids are not normally limiting for sleep.

To verify the role of glial β-oxidation in sleep-dependent lipid clearance more directly, we used *Mcad* RNAi to knockdown β-oxidation and quantified lipid droplets. Although the GS control group exhibited an increase in lipid droplets at ZT18, glial *Mcad* RNAi abolished this time point-dependent difference (Fig. 5e, comparisons at top.) Comparing between the GS control and glial *Mcad* RNAi groups, we observed that glial *Mcad* RNAi results in increased lipid droplets at ZT0 with and without sleep deprivation and at ZT6 (Fig. 5e, comparisons at the bottom), similar to glial *Drp1* RNAi and *GLaz* RNAi phenotypes. Because MCAD is specific to β-oxidation and does not have other functions like GLaz and Drp1, this indicates that glial β-oxidation is necessary for lipid droplet clearance during sleep.

Because β-oxidation itself can produce mitochondrial oxidative stress, we also quantified glial mito-ro-GFP2-Grx1 oxidation in the presence of glial *Mcad* RNAi to reduce β-oxidation. We found that the GS control group exhibits an increase in glial mito-ro-GFP2-Grx1 oxidation following sleep deprivation, as expected, whereas glial *Mcad* RNAi does not exhibit a wake-dependent increase in oxidation and instead has reduced oxidation during sleep periods at ZT6 and ZT18 (Fig. 5f, within-group comparisons at the top). However, there are no significant differences between the GS control and glial *Mcad* RNAi groups at any time points tested (Fig. 5f, between-group comparisons at the bottom). Together these results indicate that glial β-oxidation regulates glial oxidative stress in a time-dependent fashion and may contribute to, but does not fully explain, the increase in glial mito-roGFP2-Grx1 oxidation that we observe with wake.

### Sleep is required for mitochondrial turnover

**Fig. 6:**
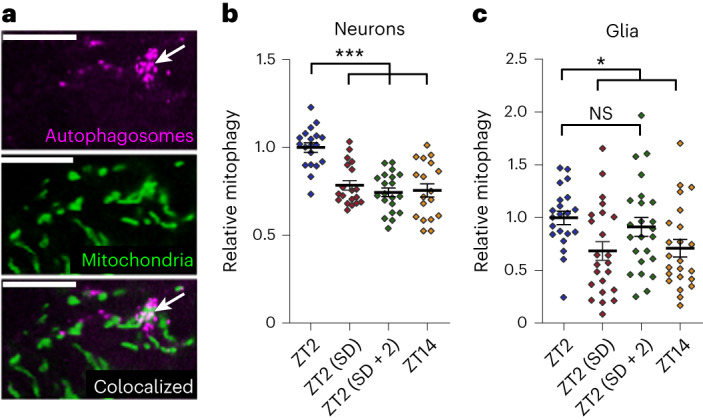
Sleep is required for mitophagy in neurons and glia. **a**, The mitochondrial and autophagy markers UAS-mito-GFP (middle) and UAS-Atg8-mCherry (top), respectively, were expressed in neurons with *nSyb*-GAL4 or glia with *repo*-GAL4. Colocalization (bottom) between autophagosomes and mitochondria was used to quantify mitophagy. The example images shown are from a small section of a single *z* slice of a brain with glial marker expression. The arrow indicates colocalization between mitochondria and autophagosomes (mitophagy); scale bars, 10 μm. **b**,**c**, Neuronal (**b**) and glial (**c**) mitophagy are greatest in the morning (ZT2) following a full night of sleep. Reduced mitophagy following a day of wake (ZT14) or a night of sleep deprivation (ZT2 (SD)) indicates that changes in mitophagy are sleep dependent rather than circadian clock dependent. Sleep deprivation/recovery and circadian time of fly collection are the same as shown in Fig. 1. For all data shown, *P < 0.05, **P < 0.01 and ***P < 0.001, while some groups with *P* > 0.05 (not significant) are unmarked. Bars/error bars indicate mean and s.e.m., respectively. Data points indicate individual brains/flies. The following are the numbers of flies (*n*) as plotted from left to right and statistical tests used: *n* = 19, 19, 20 and 18, Kruskall–Wallis test with a Dunn’s multiple testing correction (**b**); *n* = 21, 23, 24 and 23, Kruskall–Wallis test with a Dunn’s multiple testing correction (**c**). Source data

Our data indicate that optimal Drp1-dependent activity targeting both neuronal and glial mitochondria is required for the lipid production and catabolism that drives normal levels of daily sleep. A major function of Drp1 is the promotion of mitochondrial fission to facilitate selective mitophagy of damaged sections of mitochondria while sparing healthy sections^53,54^. There is strong evidence that sleep loss results in accumulation of neuronal mitochondrial damage^10,55,56^, but it is not clear if mitochondrial turnover helps to alleviate this damage. To assess the dependency of macromitophagy^57^ in neurons and glia on sleep, we quantified colocalization of the autophagic marker Atg8a-mCherry with mito-GFP-labeled mitochondria (Fig. 6a) across different sleep and circadian time points (as in Fig. 1a).

We found that Atg8a-dependent mitophagy was higher at ZT2 following a night of sleep than at ZT14 following a day of wake in both neurons and glia. Dependence on sleep, and not just time of day, was indicated by the reduction in mitophagy at ZT2 following a night of sleep deprivation. Neuronal mitophagy required a full night of sleep for induction, whereas glial changes were more variable and appeared to require less sleep for induction, with increases apparent after just 2 h of recovery sleep (Fig. 6b,c). These results demonstrate that sleep is required for maximal levels of glial and neuronal mitophagy.

## Discussion

**Fig. 7:**
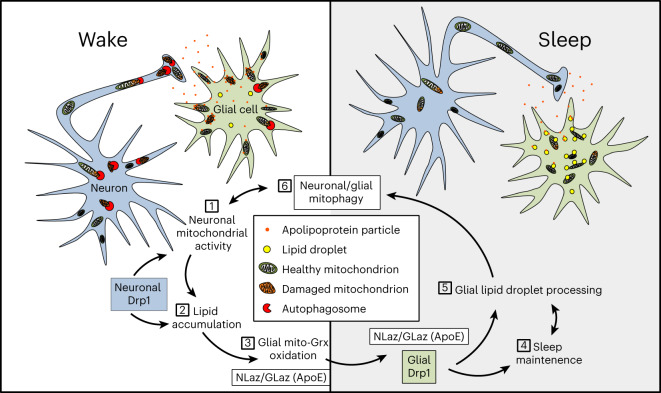
Model for a sleep-regulated metabolic cycle between neurons and glia. (1) During wake, mitochondrial energetic activity in neurons results in the production of lipids, which are transferred to glia. (2) As lipids are transferred during wake, glial mitochondria become oxidized, as indicated by mito-roGFP-Grx1 and MitoTimer. Neuronal *Drp1* (blue) is required for maximal mitochondrial energetic efficiency and subsequent glial lipid accumulation. (3) Wake-driven glial mitochondrial oxidative stress and lipid accumulation also require the expression of the lipid transfer genes *GLaz* in glia and *NLaz* in neurons. Reductions in *GLaz* or *NLaz* disrupt lipid droplet dynamics and cause mitochondrial oxidative stress to accumulate in neurons rather than in glia (Fig. 3e). Sustained lipid delivery and subsequent catabolism of lipids in glia, facilitated by neuronal NLaz, glial GLaz, Drp1 and glial mitochondrial β-oxidation (*Mcad*), are required for daily sleep (4), and, in turn, sleep is necessary for glial lipid catabolism (5). Finally, sleep promotes mitophagy in neurons and glia (6), ensuring the maintenance of a new/healthy population of mitochondria, which are critical for maximally efficient neuronal mitochondrial activity during a new day of wake (1).

Brain energetic limits are defined not only by the availability of fuels but also by the ability of the brain to use these fuels without generating cellular damage that exceeds repair capacity. Although mitochondrial oxidative phosphorylation is the fastest and most efficient way for cells to produce energy, it necessitates constant surveillance of mitochondrial health to prevent inefficient substrate use and broader oxidative damage, which is especially important to highly sensitive postmitotic neurons. We showed here that glia accumulate mitochondrial oxidative stress with wake and lipid droplets during subsequent sleep in a manner dependent on the *Drosophila* *APOE* orthologs *GLaz* and *NLaz*. Knockdown of *NLaz* or *GLaz* disrupts wake-driven glial lipid droplet accumulation and shifts mitochondrial oxidative stress from glia to neurons. Further, linking these findings to sleep, we found that knockdown of *GLaz*, *NLaz*, glial β-oxidation genes or genes responsible for the rectification of mitochondrial damage via selective mitophagy (*Drp1*, *Pink1* and *park*) resulted in sleep loss and fragmentation. In turn, sleep also promoted lipid catabolism and mitophagy, thus assuring the recovery of mitochondrial energetic efficiency (Fig. 7). Based on our findings, we propose that essential functions of daily sleep include the protection of neuronal mitochondria from oxidative damage and the maintenance of neuronal energetic efficiency, which are both driven by mitophagy and neuron–glia lipid turnover. This cycle supports high levels of neuronal mitochondrial activity while minimizing neuronal oxidative damage resulting from energy production.

We have shown here that the accumulation of lipid droplets is dependent on prior wake, occurring at ZT2 following sleep deprivation or at ZT6, ZT14 and ZT18 during sleep periods following daily wake (Fig. 2a,b,d). Accumulation varies by genotype at ZT6, likely due to variations in daytime sleep, but occurs consistently at ZT14–Z18. Variability is also seen at peak wake time points (ZT0 with sleep deprivation and ZT12) that are under strong circadian control. Conversely, glial mito-roGFP2-Grx1 oxidation, while it is also dependent on prior wake, is most consistently increased at peak wake time points (ZT0 + sleep deprivation and ZT12) but more variable during sleep periods. Because knockdown of the lipid transporters *GLaz* and *NLaz* affects both lipid droplets and mito-roGFP2-Grx1 oxidation, these differences (summarized in Extended Data Fig. 2d,e) suggest that glia may process lipids and lipid-dependent oxidative damage differently during sleep (via lipid droplets and MDA production) and wake periods (via glutathione). In addition, it is possible that increases in lipid droplets during sleep periods are the result of an increased rate of neuron–glial lipid transfer at these times or increased neuronal lipid production via autophagy, which is activated by sleep^3^.

### Lipid transfer under normal and pathological conditions

We report a daily cycle of glial lipid droplet accumulation and catabolism that is not only non-pathological but also necessary for the maintenance of brain mitochondrial health via transfer of oxidative stress from neurons to glia and sleep-dependent mitophagy. This is supported by findings showing that glial lipid droplet accumulation prevents neuronal aging by supporting glial and neuronal mitochondrial activity^19^. Previously, neuronal^7,8,19,36^ or glial^26,27^ mitochondrially driven lipid droplet accumulation in *Drosophila* was only shown to occur in the context of developmental pathology/neurodegeneration. Further, in both humans and mice, lipid droplet accumulation in microglia is associated with a transition to a proinflammatory neurodegenerative state^24,58^. We expect that these neurodegenerative results are complementary to our findings, whereby a healthy cycle of glial lipid accumulation and catabolism may become pathological if glia are overloaded with more lipids than they are able to efficiently catabolize. Importantly, our work provides a possible mechanism (loss of mitochondrial health and daily glial lipid catabolism) by which sleep loss contributes to neurodegeneration. In fact, sleep disturbances are linked to Alzheimer’s disease and are often present before other symptoms or markers^59,60^. In this vein, it is also intriguing that a specific transport-defective isoform of APOE, APOE4 (the mammalian functional ortholog of GLaz^36^), is a major risk factor for Alzheimer’s disease and that additional Alzheimer’s disease-associated genes also alter the dynamics of neuron–glia lipid metabolism^61^. Further supporting the existence of distinct healthy and pathological glial lipid processing states, human induced pluripotent stem cell-derived microglia exposed to neuronally conditioned medium induce a program of lipid uptake, oxidative phosphorylation and β-oxidation gene expression, whereas *APOE4-*knock-in microglia suppress mitochondrial gene expression and accumulate lipid droplets^58^.

Our findings strongly suggest that some of the lipids transferred from neurons to glia on a daily basis are peroxidated. First, we found that our glutathione-based oxidation sensor mito-roGFP2-Grx1, which shows wake- and lipid transport-dependent glial mitochondrial oxidation, can be fully oxidized by the lipid peroxide modeling compound cumene hydroperoxide (Fig. 1e), suggesting a sensitivity to lipid peroxides. Second, we found that brain levels of the lipid peroxide breakdown product MDA are increased during sleep at the same time points that glial lipid droplet counts and processing are increased (Fig. 2b,c). Third, we found that wake-driven mito-roGFP2-Grx1 oxidation is affected reciprocally in neurons and glia by knockdown of the genes encoding the lipid transporters NLaz and GLaz (Fig. 3b,d,e) but not significantly by knockdown of glial β-oxidation alone (*Mcad* RNAi versus the GS control (bottom); Fig. 5f), suggesting that sensor oxidation is mediated at least in part by glial uptake of extracellular lipids, independently from β-oxidation.

The well-documented roles of GLaz and NLaz in intercellular lipid transport^36,62^, as well as the reciprocal effects on glial and neuronal mitochondrial oxidation that we observed with transporter knockdown (Fig. 3a,b), clearly indicate that these proteins mediate the transport of lipids and associated mitochondrial glutathione oxidation from neurons to glia on a daily basis. Consistent with this, neuronal *NLaz* RNAi resulted in a reduction in lipid droplets (Fig. 3d). Surprisingly, however, glial *GLaz* RNAi increased lipid droplets in the first half of the day (Fig. 3c; ZT0–ZT6). Interestingly, knockdown of glial β-oxidation (*Mcad* RNAi) also resulted in excess lipid droplets in the first half of the day, when control levels were lower (Fig. 5e). Excess lipid droplets in the first half of the day are consistent with impaired lipid clearance during sleep and suggest a potential role for GLaz in lipid catabolism, which may be mediated by lipid transport within the cell.

Glia are capable of detoxifying peroxidated lipids, whereby mitochondrial β-oxidation of peroxidated lipids reduces ROS^8^. The idea that sleep serves to recycle damaged membrane phospholipids, in part via mitochondrial β-oxidation, was suggested by metabolomic profiles of the cortex in sleep-deprived mice that revealed significant levels of lysolipids and long-chain acylcarnitines (carnitine conjugation supports mitochondrial import of lipids for β-oxidation)^63^. Recently, we also found that disruption of blood–brain barrier lipid transporters and receptors in *Drosophila* affects sleep and increases long-chain acylcarnitine levels in the head. Interestingly, feeding of acylcarnitines increases sleep, suggesting that lipid metabolism has an instructive role in the generation of sleep need^64^.

*Drp1* activity is regulated by the circadian clock^65^, and the mitochondrial quality control genes *Pink1* and *park* have previously been linked to circadian rhythms and sleep structure in *Drosophila*^44,46,47^ as well as humans^66,67^. Lipid metabolism has also been linked to sleep; *Lsd-2* mutants, which have impairments in lipid droplet storage, do not display rebound sleep, whereas *bmm* mutants, which have excessive lipid storage, exhibit excessive rebound sleep after deprivation^68,69^. Fatty acid-binding proteins are known to facilitate lipid processing, metabolism, trafficking and signaling^70,71^ and are necessary for both daily and rebound sleep, and their overexpression promotes long-term memory consolidation and ameliorates sleep deficits in an Alzheimer’s disease model^72–74^. In agreement with our finding that mitochondrial β-oxidation is important for sleep, the acyl-CoA synthase Pdgy promotes sleep and is required for rebound sleep in *Drosophila*^75^. However, the mechanisms underlying all of these effects were unknown. Our findings provide an explanation for these data and incorporate them into a model for a metabolic function of sleep.

If sleep-dependent lipid catabolism in glia is important for detoxifying low levels of peroxidated lipids transferred from neurons, sleep loss should result in accumulation of oxidative damage in the brain. In fact, it is well-established that prolonged sleep loss results in oxidative cellular, DNA and mitochondrial damage^55,56,76^. In cases of sleep deprivation or artificially induced oxidative stress, mitochondrially generated ROS is capable of directly promoting recovery sleep^10^, although this mechanism pertains specifically to recovery sleep following deprivation and applies only to a small subset of sleep-promoting neurons in the *Drosophila* brain. Nevertheless, daily sleep is important for detoxifying ROS and/or preventing ROS generation, and we suggest that it occurs in a brain-wide fashion at least in part through the mechanisms that we report here.

### Mitochondrial energetic activity and quality control in sleep

*Drosophila* and most mouse *Drp1*-mutant neurons exhibit normal morphology and electrophysiological and synaptic properties but have a specific deficit in the ability to maintain high firing rates due to a reduced rate of presynaptic ATP generation^77–79^. The unique combination of deficits associated with loss of neuronal *Drp1* has provided us with a window through which to observe how non-pathological reductions in neuronal mitochondrial activity and selective mitophagy affect sleep. A reduction in neuronal mitophagy might be expected to result in reduced glial lipid accumulation and increased neuronal oxidative damage; however, loss of neuronal *Drp1* does not result in accumulation of oxidative damage in most *Drosophila* neurons (Fig. 4e), or mouse forebrain neurons^79^. The most parsimonious explanation for this is that a reduction in energetic activity in neurons lacking *Drp1* results in less oxidative damage in the first place. Thus, in neurons, the effects of reduced selective mitophagy on oxidative stress may be mitigated by reduced energetic activity. On the other hand, both of these factors could account for the reduced glial lipid accumulation that we observed with neuronal *Drp1* RNAi (Fig. 4a). *Drp1* RNAi in neurons or glia results in a decrease in sleep, despite different effects on lipid droplets. We suggest that this is because both manipulations ultimately reduce glial β-oxidation, although *Drp1* in glia may also regulate lipid droplet formation^80^.

In summary, we report a basic mechanism and function of healthy daily sleep, which could be relevant for understanding how sleep loss predisposes to neurodegenerative diseases and aging more generally. Suboptimal neuronal and glial mitochondrial energetic activity or efficiency in such cases may impair the amplitude of daily glial lipid accumulation cycles, resulting in a negative feedback of sleep loss, reduced mitochondrial turnover and further suppression of healthy mitochondrial activity. Our findings that sleep loss occurs in the absence of any oxidative damage or locomotor deficits when *Drp1* is knocked down supports the possibility that sleep loss itself is an early step leading to late-onset neurodegeneration and/or normal aging. Thus, understanding this sleep-metabolism feedback cycle may allow us to short circuit certain mitochondrial-based aging and/or disease processes.

## Methods

### Fly stocks

The following lines were obtained from the Vienna *Drosophila* Resource Center: UAS-*Drp1*-RNAi 44155, UAS-*yip2*-RNAi (*ACAA*) 26562, UAS-*Echs1*-RNAi 27658, UAS-*Mcad*-RNAi (*ACAD*) 15053, UAS-*whd*-RNAi (*CPT1*) 4046, UAS-*NLaz*-RNAi 35558 (targets exons 1–3, efficacy previously verified^36,81^), UAS-*NLaz*-RNAi 101321 (targets exon 1 only, no sleep loss with *nSyb*-GS on RU-486, previously only used in larvae^82^), UAS-*GLaz*-RNAi 15389/15387 (same construct different insertion sites, efficacy previously verified^36,81^), UAS-*GLaz*-RNAi 107433 (embryonic lethal when crossed with *repo*-GS in the absence of RU-486), *Pink1*-RNAi 21860, *park*-RNAi 47636 and *Miro* shRNAi 330334.

The following lines were obtained from the Bloomington *Drosophila* Stock Center: UAS-*Drp1*-RNAi 51483, UAS-MitoTimer 57323, R23E10-GAL4 (dFSB) 49032, UAS-mCherry-Atg8a 37750, UAS-mito-HA-GFP 8443 (outcrossed to Iso31 to remove the ebony marker), UAS-*Dcr-2* 24650, *pTub*-GAL80ts 7019/7017, UAS-mito-roGFP2-Grx1 67664, *Marf* RNAi 67158 and *GLaz* RNAi 67228.

The following lines were obtained from other sources: UAS-*Drp1* (K38A (ref. ^48^); gift from D. Walker, UCLA), *nSyb*-GAL4, *repo*-GAL4, Gad2B-GAL4 and white Canton-S (gifts from L. Griffith, Brandeis), UAS-dTrpA1 (gift from P. Garrity, Brandeis), *repo*-GS^5^ (gift from H. Tricore, Paris Diderot University), R26E01(A)-GAL4 (gift from G. Rubin, Janelia), 9-137-GAL4 (ref. ^49^; gift from R. Bainton, UCSF), *Eaat1*-GAL4, NP2222-GAL4, MZ0709-GAL4 (ref. ^33^) (gifts of M. Freeman, OHSU), 2×(UAS-GFP-*Lsd-2*)^34,35^ and UAS-LD-GFP (Klar lipid droplet-targeting domain^29^; gifts from M. Welte, University of Rochester). CaLexA flies (w; UAS-CD8::RFP, LexAop-CD8::GFP-2A-CD8::GFP; UAS-mLexA-VP16-NFAT, LexAop-CD2::GFP) were a gift from J. W. Wang, UCSD^31^. The following lines are from the Sehgal lab stocks: *nSyb*-GS^83^, *ple*-GAL4 and *Act5C*-GS^84^.

Experimental and control genotypes used are indicated in the figure legends. Because mito-roGFP2-Grx1 requires GS/GAL4 for expression, it was not possible to have a UAS control group for these experiments (Figs. 3a,b, 4e and 5f). Because lipid droplets were quantified from the same set of experiments in some instances, they also do not have a UAS-alone control group (Figs. 3c,d, 4e and 5e).

### Fly rearing and RU-486

Flies were maintained on a 12-h light/12-h dark cycle at all times during rearing and testing. All crosses were raised and maintained at 25 °C, except glial subset-GAL4, *pTub*-GAL80ts and *nSyb*>dTrpA1 crosses, which were maintained at 18 °C (pTub-GAL80ts) or 23 °C (nSyb>dTrpA1) and shifted to either 31 °C or 25.5 °C, respectively, for activation. In all experiments, mated female flies were collected 1–5 d after eclosion (generally within a 3-d age range), with data collection beginning at 4–7 d after eclosion. Data were not used beyond 14 d after eclosion, with the exception of Extended Data Fig. 4e for geotaxis and survival data with 10 d on RU-486 (ending at 14–17 d after eclosion). In UAS-GAL4/GS experiments, the respective UAS and GAL4/GS lines were crossed to white Canton-S flies to generate heterozygous control groups. All experimental lines for RNAi experiments contained UAS-*Dcr-2* to promote RNAi efficiency. Flies were raised and entrained on standard cornmeal molasses food. In experiments with GeneSwitch-GAL4 lines (GS), flies were transferred at 3+ d after eclosion to 5% sucrose/2% agar food containing 100 μM or 500 μM mifepristone (RU-486). When lifespan experiments indicated that 500 μM RU-486 reduced the lifespan in all genotypes, the dosage was adjusted to 100 μM for all subsequent experiments, which we found to result in equivalent behavioral sleep phenotypes. This issue has been documented elsewhere^85^. All experiments in all main and Extended Data figures using RU-486-driven induction (with the exception of Extended Data Fig. 4e) were completed within 6 or fewer days of initial RU-486 induction on sucrose agar food. In experiments with RU-486, all experimental and control groups were transferred to and maintained on RU-486-containing food identically. For experiments in Extended Data Fig. 5a–c, all (–) RU-486 groups had equal amounts of ethanol (RU-486 solvent) in the food.

For mito-roGFP2-Grx1, MDA and lipid droplet experiments, to reduce variability, flies were flipped to fresh molasses food or RU-486-containing sucrose agar each day for a minimum of 2 d before dissection. Before implementing this daily flip procedure, we occasionally observed very high levels of mito-roGFP-Grx1 sensor oxidation in our healthy control groups. In these same experiments, if RU-486 was used, flies were transferred to RU-486 48–72 h maximum before dissection.

### Sleep behavior

Mated female flies, 3–6 d old, were loaded into 65 mm × 5 mm glass locomotor tubes containing 5% sucrose/2% agar food (including RU-486 for GeneSwitch experiments). Flies were always given a minimum of 1 d of acclimation before collection of data to be used in experimental analysis. Flies were maintained on a 12-h light/12-h dark cycle at 25 °C (with the exception of *nSyb*>dTrpA1 and *pTub*-GAL80ts glial subset-GAL4 experiments, which had temperature shifts, as indicated previously). Sleep and locomotor activity data were collected in 1-min bins using the *Drosophila* Activity Monitoring (DAM) System (TriKinetics). Sleep was defined as bouts of uninterrupted inactivity lasting for ≥5 min^86,87^. Sleep/activity parameters (total sleep, mean sleep episode duration, number of sleep episodes and activity while awake) were analyzed for each 24-h period and averaged across 2 or more days (excluding the first day to allow for acclimation). Sleep analysis was conducted using the MATLAB program SCAMP, as described previously^88^. To simplify data presentation and allow comparison across experimental groups, sleep data are presented as the difference between experimental and control groups. These values were calculated by subtracting the value for each experimental fly from the mean of the control group. Error was propagated in s.e.m. error bars for all such figures. Statistical comparisons were performed on raw (not subtracted) data. All sleep experiments were replicated with statistically significant results at least once. Data shown in plots and figures are from a representative experiment.

### Sleep deprivation

Sleep deprivation was performed using a vortexer from Trikinetics with shaking for a random 2 s of every 20 s. For mitophagy, mito-roGFP2-Grx1, MDA and lipid droplet experiments, flies were entrained and sleep deprived in vials of 30 or fewer flies per vial taped to the vortexer. In all other experiments, flies were sleep deprived in glass DAM locomotor tubes. All flies were sleep deprived from ZT12 or ZT14 of the previous night up until removal for dissection. The sleep deprivation + recovery group was sleep deprived from ZT14 to ZT0, removed from the shaker at ZT0 and allowed to sleep until ZT2, as indicated in Fig. 1a. If sleep recovery in the first 2 h of the morning was allowed, collection times for all groups were shifted by 2 h (Fig. 1a, filled diamonds).

### Fatty acid feeding

Fatty acids were added at 10% (wt/vol) to 5% sucrose/2% agar food containing 100 μM RU-486. An equal volume of water was added to the control food so that sucrose and RU-486 concentrations were equal across groups (this results in a final concentration of 4.5% sucrose and 90 μM RU-486 for all groups). Fats that are solid at room temperature (palmitate and stearate) were first melted in Falcon tubes in a hot water bath before mixing with sucrose/agar food. Because fats are not soluble in sucrose/agar food, liquid fat and sucrose/agar food were combined while hot and quickly vortexed to emulsify before pouring/cooling. Palmitate (10006627) and stearate (10011298) were purchased from Cayman Chemical. The olive oil used was Filippo Berio brand organic extra virgin olive oil.

### Lifespan and negative geotaxis

Three- to 7-d-old mated female flies were sorted into groups of ten flies each (four groups per genotype) and transferred to vials containing 5% sucrose/2% agar + 100 μM RU-486. Flies were flipped to fresh food every 4 d, with the number alive and negative geotaxis (percent climbing) quantified the day after flipping. Negative geotaxis was quantified by tapping the vial five times to knock all flies to the bottom and counting the percentage of live flies able to climb above a mark 2 cm from the bottom of the vial within 10 s. Percent climbing values for each vial are the average of two trials on each day of testing. All testing was between ZT2 and ZT6. Flies were maintained at 25 °C on a 12-h light/12-h dark cycle at all times.

### qPCR

UAS-*Drp1*-RNAi 44155 or UAS-*Pink1*-RNAi 21860 were crossed to *Act5C*-GS, and 3- to 5-d-old mated female and male progeny were collected. Flies were transferred to 5% sucrose/2% agar food containing 500 μM RU-486 for 5–6 d before RNA extraction. RNA extraction was performed on whole flies (5–15 flies per genotype/experiment, with a total of four biological replicate experiments). RNA was extracted using TRIzol (Fisher, 10296010) and reverse transcribed to cDNA using random hexamers and Superscript II (Invitrogen, 18064014). Real-time PCR was performed using Sybr Green (Applied Biosystems) on a ViiA7 Real-Time PCR machine (Applied Biosystems). The change in cycling threshold (ΔΔ*C*_t_) method was used to quantify gene expression as normalized to *Act79B*. All primers are shown in Table 1.

**Table 1:** Primers used for qPCR

Gene	Primer (5′–3′)
*Drp1*-forward	GACTCCATCCAATTGCCCCA
*Drp1*-reverse	TGGACGTACCATTTTCCGCC
*Pink1*-forward	TCTTAAAGAATAGTTGCAGGCAC
*Pink1*-reverse	TGGTCCAAAATGTTGGCGTG
*Act79B*-forward	CTGGCGGCACTACCATGTATC
*Act79B*-reverse	GGACCGGACTCGTCATACTC

### Immunofluorescence, animal/sample preparation and microscopy

Mitophagy and MitoTimer imaging was performed on a Leica SP5 confocal microscope, mito-roGFP2-Grx1 experiments were performed on a Leica SP8 confocal microscope, and lipid droplet and MDA imaging was performed on a Leica SP5, SP8 or Leica Stellaris confocal microscope. Sequential acquisition was always used when imaging at multiple excitation and/or emission wavelengths to avoid any bleed through between excitation/emission spectra.

MitoTimer imaging was performed on live ex vivo brains with a ×40 water immersion objective. Entrained flies were first loaded into sucrose/agar-containing glass DAM locomotor tubes, as in the sleep experiments, and were allowed to acclimate for ≥1 d before sleep deprivation and testing. All dissection and imaging experiments were performed between ZT0 and ZT2, alternating between control and sleep-deprived groups during this time. Mechanical sleep deprivation occurred beginning at ZT14 the previous night and continued throughout the imaging period up until dissection. Flies were removed, dissected and imaged individually with brief anesthesia on ice before dissection. Dissection and imaging were performed in artificial hemolymph solution (AHL)^89^. Green fluorescence was imaged with excitation/emission at 488/496–537 nm, and red fluorescence was imaged with excitation/emission at 543/560–620 nm.

Mitophagy, MDA and lipid droplet (Nile Red/BODIPY 493/Lsd-2–GFP/LD–GFP) experiments were performed on fixed brains. A 16% formaldehyde solution (Electron Microscopy Sciences, 15710) was immediately aliquoted after opening and stored at −20 °C until use. Dissected brains were fixed for 20 min at room temperature in 4% formaldehyde diluted in PBS followed by a wash in PBS. To expedite fixation for mitophagy experiments, whole heads with the proboscis removed were fixed before brain dissection and mounting. Brains were mounted in Vectashield (Vector Labs, H-1000) before imaging of central brains with a ×63 oil immersion objective. All groups to be compared were always imaged together on the same day. Brains with Lsd-2–GFP or LD–GFP were stained overnight at 4 °C with rabbit polyclonal anti-GFP (1:1,000; Invitrogen, A-11122). MDA antibody staining (Sigma mouse monocolonal 11E3, 1:50, SAB5202544) was also conducted overnight at 4 °C. Primary and secondary antibody incubations were performed in PBS + 0.3% Triton X-100 (PBT). Following primary antibody staining, brains were washed three times for 5 min each in PBT, stained for 3 h with Alexa 488 anti-rabbit (1:500; Fisher, A-11008), washed three times for 5 min each in PBS, mounted and imaged. Goat serum (10%) was added to PBT for blocking during all antibody staining steps.

For mitophagy experiments UAS-mito-GFP was used to label mitochondria, and UAS-mCherry-Atg8a was used to label autophagosomes. Neuronal expression was driven by *nSyb*-GAL4, and glial expression was driven by *repo*-GAL4. We found antibody staining unnecessary and used the endogenous fluorescence of mito-GFP (excitation/emission: 488/500–537 nm) and mCherry-Atg8a (excitation/emission: 543/555–632 nm) for image acquisition. All neuronal images and analyses encompass the entire central brain (excluding optic lobes) acquired as 80 × 2 μm *z* slices (1,024 × 1,024 pixels) for nSyb images. Glial images and analyses represent the anterior half of the central brain with 30 × 2 μm *z* slices (1,024 × 1,024 pixels).

For BODIPY and Nile Red lipid droplet staining, we found it critical that the dyes (BODIPY 493/503, Fisher-D3922; Nile Red, Fisher-N1142) were aliquoted in freshly opened/anhydrous DMSO (Fisher, D12345) and frozen immediately at −20 °C or −80 °C without any freeze–thaw cycles before use. For staining, 1 or 5 mg ml^−1^ aliquots were diluted 1:1,000 (for final concentrations of 1 μg ml^−1^ for BODIPY 493 and 5 μg ml^−1^ for Nile Red) in PBT and vortexed. In early experiments (Fig. 2b at ZT2 versus ZT14 only, Figs. 2d and 4a,b and Extended Data Fig. 2g), fixed brains were stained for 10 min in 0.3% PBT at room temperature, followed by a wash in PBS and same-day mounting and imaging. In later experiments (Figs. 2b (ZT0, ZT6, ZT12 and ZT18), 3c,d, 4e and 5e), we found that overnight incubation at 4 °C in 0.3% PBT before a 10-min incubation with BODIPY 493 or Nile Red dyes further improved brain lipid droplet staining (reduced background and improved dye penetration); however, it was critical that brains be transferred directly from dye to Vectashield (no wash) to prevent dye washout. In addition, we found that PBT concentrations at or exceeding 0.5% Triton X-100 resulted in disruption of lipid droplet structure, as evidenced by fragmentation of Lsd-2–GFP rings. For all lipid droplet experiments, Nile Red/BODIPY 493-stained central brains were imaged at ×40 or ×63, 2,048 × 2,048 pixels, with 30–40 × 2 μm *z* slices per brain. The number of *z* slices imaged per brain was held constant within each experiment, and all data were normalized to the ZT0–ZT2 control before pooling across experiments.

To better preserve endogenous mito-roGFP-Grx1 sensor fluorescence, all brains were fixed in 2% formaldehyde + 20 mM *N*-ethyl maleimide (NEM) in PBS for 1 h at room temperature. NEM (Sigma, E3876) is a fast-acting roGFP2-thiol-blocking agent^14^ that prevents further probe oxidation or reduction. We verified that the function of mito-roGFP2-Grx1 is maintained in fixed tissue, as previously reported^14^. Before fixation, flies were anesthetized on ice and dissected in AHL (Fig. 1c–f and Extended Data Fig. 1a–c). For experiments in Fig. 1c,d, brains were dissected and stored on ice in AHL + 20 mM NEM until fixation. Because we have found that Schneider’s medium (Fisher, 21720024) promotes maximal levels or respiration in *Drosophila* brains, this rich medium was used in control experiments in Fig. 1e,f and Extended Data Fig. 1a–c to promote rotenone-dependent mitochondrial ROS generation. For experiments in Fig. 1e,f and Extended Data Fig. 1a, brains were incubated in Schneider’s medium containing the indicated concentrations of rotenone at ~ZT6 for 2 h at room temperature. For the cumene experiment in Fig. 1e, brains were incubated at room temperature for 30 min in 2 mM cumene hydroperoxide (Sigma, 247502) in AHL. DA (5 mM; Sigma, D3648) or DTT (5 mM; Fisher, R0861) incubations were performed for 30 min on ice. Before fixation, all brains were incubated in 20 mM NEM in AHL at room temperature for 10 min. All mito-roGFP2-Grx1 brains were mounted in Vectashield and imaged on a Leica SP8 confocal microscope with a ×63 immersion objective, as indicated above. Light at 405 or 488 nm was used for excitation, whereas the emission range was held constant at 495–520 nm. We note that it is very important to keep the imaging room and microscope at a constant temperature for imaging with this sensor.

For CaLexA imaging, adult male fly brains were dissected in cold PBS and fixed for 40 min in 2% paraformaldehyde at room temperature. The brains were then washed for 20 min twice in PBS supplemented with 0.3% Triton X-100. Brains were then transferred into glycerol and mounted with Vectashield. Endogenous GFP and red fluorescent protein (RFP) signals were visualized without secondary amplification of endogenous signal with antibodies. Both control and experimental brains were mounted on the same slides, and identical settings were used for the laser intensity and gain. After image acquisition on a Leica Stellaris confocal microscope, Fiji/ImageJ was used for image analysis. CaLexA data are presented as GFP:RFP ratios of total signal from *z*-projection images.

### Image analysis

For all representative images, for visualization purposes, brightness and contrast were adjusted identically within each group. In Fig. 1d, images were created by dividing the summed 405-nm and 488-nm images on a pixel-by-pixel basis and false coloring with the fire LUT in ImageJ. If the fire LUT was used, the color calibration bar was included. For representative images in Fig. 2a,c, we note that spatial localization and labeling patterns were highly variable from one brain to the next. Therefore, the representative images shown are representative only of relative MDA intensity or lipid droplet count differences and are not representative of any particular spatial localization pattern. Original/raw image files have been provided in supplemental files for all representative images used in figures.

In all image analysis experiments, all brains were normalized within each experiment to the mean of the ZT0/ZT2 or UAS control group before pooling data across repeated experiments. Statistical differences within each experiment were then calculated by comparing each group to the ZT0/ZT2 control with appropriate multiple comparisons tests.

For analysis of all MitoTimer and mito-roGFP2-Grx1 images, analyses were conducted with a custom macro in ImageJ using methods based on a previous report^13^. First, to exclude background and debris/autofluorescent trachea, images were divided on a pixel-by-pixel basis, and any pixels outside of a set range of intensities were excluded. After exclusion of ratio-outlier pixels, any remaining background was removed from each channel individually by excluding pixels with values less than a constant multiplied by the mean of that channel. For all mito-roGFP2-Grx1 brains, pixels with a 405:488 ratio less than 1:4 or greater than 4:1 were excluded (that is, excluding all pixels outside the range of 0.25×–4×), and to remove the remaining background, pixels less than 0.5× the mean of each channel were also excluded. For MitoTimer, because each set of experiments had very different levels of signal and background intensities (resulting from differences in driver expression strength, image acquisition settings and live-imaging-induced light scatter), ratios and constants were optimized for each experimental set individually to include as much signal as possible while excluding background/debris. All image acquisition and analysis parameters were the same for replicates within each set of experiments. MitoTimer analysis parameters for each set of experiments were as follows: *repo* sleep deprived versus non-sleep deprived controls included ratio pixels within the range of 1/2.5×–2.5× and greater than 0.4× the channel mean; *nSyb* sleep deprived versus non-sleep deprived controls included ratio pixels within the range of 1/4×–4× and greater than 0.25× the channel mean. For both MitoTimer and mito-ro-GFP-Grx1, before pooling results across multiple experimental days, all values were normalized to the mean of the control group for a given experimental day. To convert the raw normalized data in Fig. 1e,f into percent oxidized in Extended Data Fig. 1a, the following equation was used: percent oxidized = (*x* – DTT)/(DA – DTT), where *x* is the control-normalized value for the respective brain, and DA and DTT are the average DA or DTT values in the respective cell type from the same experiment. In other words, percent oxidized = (*x* – minimum)/(maximum – minimum). This same percent/relative oxidation calculation (equation expressed in a different form) has also been used by others^90^.

For mitophagy and early BODIPY lipid droplet analysis, images were processed to separate signal from background with the machine learning, pixel classification software ilastik^91^. We trained the pixel classification algorithm by marking greater than or equal to six different instances of signal and background on representative images from each experimental and control group, allowing the algorithm to use all features at all pixel bin sizes for classification. All training and processing for mito-GFP, mCherry-Atg8 and BODIPY images were independent. Binary images resulting from pixel classification were then analyzed in ImageJ^92^. We have previously verified that this method works for the quantification of autophagosomes^89^. The ilastik pixel classification method was used for binarization of data shown in Fig. 2b at ZT2 versus ZT14 only and in Figs. 2d and 4a,b and Extended Data Fig. 2g. We later switched to using a custom macro in ImageJ for binarization, which reduced variability. The ImageJ macro was used for analysis of data shown in Figs. 2b (ZT0, ZT6, ZT12 and ZT18), 2f, 3c,d, 4e and 5e.

For MDA images, any stained non-brain tissue, nerves or debris was first deleted from individual image slices manually. For MDA image quantification, all slices within a *z* stack were summed for each brain, and a mean was calculated for all pixels above a set background exclusion threshold. The background exclusion threshold was held constant for all brains/genotypes within an experiment but varied as needed between experiments conducted on different days.

For BODIPY lipid droplet analysis, any stained non-brain tissue or debris was first deleted from individual image slices using a custom ImageJ macro, and any additional debris was then deleted manually. For experiments with ilastik analysis, a maximum *z* projection was created, and ilastik was then run on this maximum projection to generate a binary image marking lipid droplets. For experiments using the custom ImageJ macro, the triangle (Nile Red or BODIPY 493) or Renyi Entropy (Lsd-2–GFP) autothreshold functions were used on individual slices to generate a binary image stack marking lipid droplets. For both ilastik or autothreshold methods, the ‘Analyze Particles’ function in ImageJ was then used to quantify the number of lipid droplets and the relative total area of the image covered by lipid droplets. Average lipid droplet size for each brain was calculated by dividing the total lipid droplet area by the lipid droplet count.

For mitophagy analysis, ilastik was run on full image stacks, generating binary output stacks for both mitochondrial and autophagosome images. In ImageJ, the ‘AND’ function in the image calculator was then used to create a new image stack marking only pixels containing both mitochondrial and autophagosome signals (colocalization). The total area of colocalized pixels was normalized to the total area of mitochondrial pixels for each *z* slice and summed across all *z* slices for each brain. All values were then normalized to the mean of the ZT0 group before pooling across experiments. For brains with glial expression, because the majority of signal is at the surface of the brain, the *z* slice with the greatest mito-GFP signal area was used as the starting slice for analysis (this was usually within the first five slices acquired). This allowed us to begin analysis at the same relative position for each brain while keeping the number of slices analyzed constant at 30 and reduced variability of the results.

### Seahorse

The heater on an XFe96 Seahorse Analyzer was turned off 2–24 h before running *Drosophila* experiments to allow the system to cool down to near room temperature. Port A of a fresh sensor cartridge was loaded with 20 μl of Schneider’s medium + 10× rotenone. The Seahorse Analyzer was then calibrated with the sensor cartridge for ~1 h before loading of *Drosophila* brains. Eighty brains from 3- to 8-d-old mated female flies were dissected between ZT0 and ZT4 and kept in Schneider’s medium on ice. Once all brains were dissected, they were loaded into an XFe96 Spheroid plate (Agilent, 102905-100) containing 180 μl of Schneider’s medium per well and carefully sunk to the bottom center of each well using forceps. The Seahorse Analyzer was set to a mix/wait/measure cycle of 1 min/30 s/3 min. Baseline OCR was recorded for 6 cycles (27 min) before rotenone injection, and OCR was then recorded for an additional 32 cycles (2 h and 24 min). OCR values for each brain were normalized to the OCR values at the end of the baseline period. Sudden increases or decreases in OCR can occur as a result of mixing when tissue moves out of the center of the well away from the sensor probe. Brains with a >30% change in OCR between two consecutive measurements were excluded from analysis. In addition, any brains in wells where drug injection failed (as evidenced by observing uninjected drug in the sensor cartridge after the experiment) were also excluded. For the experiment in Extended Data Fig. 1b,c, 1 of 80 brains was excluded due to a sudden change in OCR, and 1 of 80 brains was excluded due to drug injection failure.

### Statistics

In all experiments with a GAL4 and UAS control, the experimental group was statistically compared to each control group separately using individual unpaired *t*-tests or Mann–Whitney tests. Only the most conservative/numerically greatest *P* value of these two comparisons is reported in such cases. If the experimental group was significantly different from only one of the two controls but not the other, no difference is reported. For sleep experiments where data are plotted as experimental values minus the mean control value, all statistical comparisons were performed on raw (not subtracted) data. Sample sizes in each experiment were based on the number of samples that we were able to collect/dissect/process within 2 h while including all groups to be statistically compared to one another. Except for sleep, where representative experiments are shown, all replicates have been pooled, and all data are shown. Animals were assigned to groups based on genotype. Assignment of animals within genotype to sleep/circadian groups was random. Sample processing order and animal placement in incubators were evenly distributed whenever possible. Blinding was not used, but all analyses were performed with unbiased computer programs. The only exception to this was manual clearing of any remaining debris from brains stained for lipids droplets after prior automated clearing using a macro in ImageJ. For sleep/activity experiments, flies were excluded if they died before the end of the experiment. For imaging, damaged brains or those with weak/absent fluorescence (due to loss of mounting medium from the edge of slides in a few experiments) were excluded from analysis.

Non-parametric Mann–Whitney/Wilcoxon rank-sum tests were used for analysis of all sleep data (in MATLAB). All other statistical comparisons were performed in GraphPad Prism. First, results were tested for normality using the Shapiro–Wilk normality test. If all groups to be compared passed the normality test and showed no significant difference in variances, a *t*-test (individual comparisons) or ANOVA followed by a Holm–Sidak multiple comparisons test were used. If any group within an experiment did not pass the normality test, all statistical comparisons were performed using the Mann–Whitney test (individual comparisons) or Kruskall–Wallis test followed by a Dunn’s multiple comparisons test. DA/DTT/cumene versus controls were each compared independently in Fig. 1e. Error bars in all figures represent s.e.m. Negative geotaxis and survival data were analyzed using two-tailed, unpaired *t*-tests. For experiments including multiple different time points with or without sleep deprivation, all comparisons were performed versus the ZT0/ZT2 control group. Multiple comparisons corrections were used for multiple circadian time points, while sleep deprivation was considered an independent experiment. All statistical tests used were two sided. The numbers of animals used and specific statistical tests for each experiment/comparison are indicated in the figure legends and are shown in Extended Data Table 1.

### Reporting summary

Further information on research design is available in the Nature Portfolio Reporting Summary linked to this article.

## Online content

Any methods, additional references, Nature Portfolio reporting summaries, source data, extended data, supplementary information, acknowledgements, peer review information; details of author contributions and competing interests; and statements of data and code availability are available at 10.1038/s41593-023-01568-1.

## Supplementary information

Reporting Summary

## Source data

Source Data Fig. 1 Unprocessed images.

Source Data Fig. 1 Statistical source data.

Source Data Fig. 2 Unprocessed images.

Source Data Fig. 2 Statistical source data.

Source Data Fig. 3 Statistical source data.

Source Data Fig. 4 Statistical source data.

Source Data Fig. 5 Statistical source data.

Source Data Fig. 6 Unprocessed images.

Source Data Fig. 6 Statistical source data.

Source Data Extended Data Fig. 1 Statistical source data.

Source Data Extended Data Fig. 2 Unprocessed images.

Source Data Extended Data Fig. 2 Statistical source data.

Source Data Extended Data Fig. 3 Unprocessed images.

Source Data Extended Data Fig. 4 Statistical source data.

Source Data Extended Data Fig. 5 Statistical source data.

Source Data Extended Data Fig. 6 Statistical source data.

Source Data Extended Data Fig. 7 Statistical source data.

## Figures and Tables

**Extended Data Table 1:**
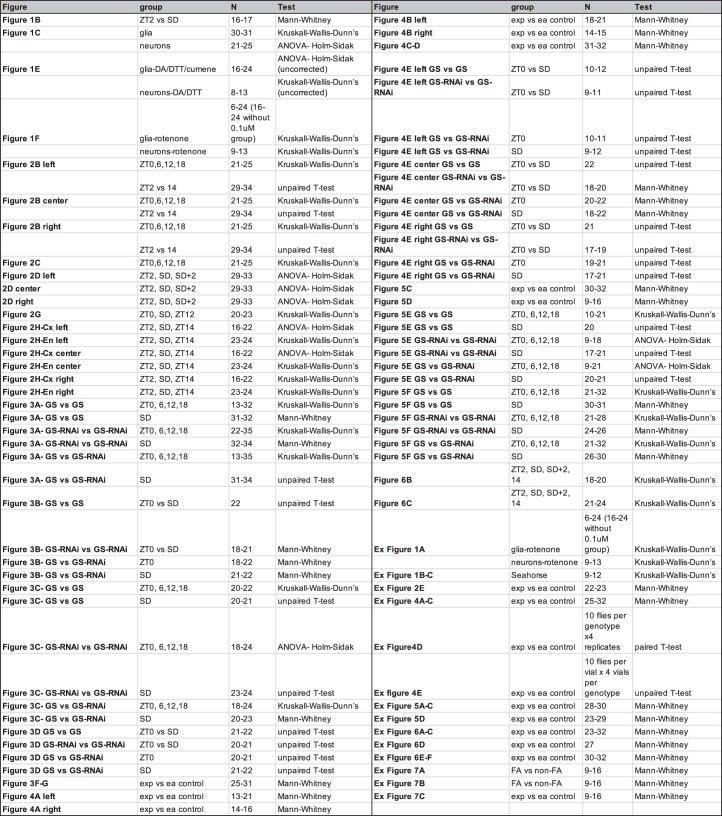
Number of animals (N) and statistical tests used for each experiment

## Data Availability

Source data are provided with this paper. Source image files are also available on Figshare at 10.6084/m9.figshare.24905223.

## Code availability

ImageJ/Fiji image analysis software can be accessed at http://fiji.sc.

Ilastik image analysis software can be accessed at https://www.ilastik.org.

SCAMP MATLAB code used for sleep analysis is available at the Vecsey lab website at https://academics.skidmore.edu/blogs/cvecsey/.

ImageJ macros use built-in functions, as indicated in the Methods, and can be shared upon request.
