# Supplementary material for: A neuron–glia lipid metabolic cycle couples daily sleep to mitochondrial homeostasis
Source: Nat Neurosci. 2024 Feb 15;27(4):666–78. doi: 10.1038/s41593-023-01568-1 (PMC11001586; doi:10.1038/s41593-023-01568-1)
Supplement: Supplementary file 1 — Reporting Summary [file 41593_2023_1568_MOESM1_ESM.pdf]

nature portfolio

Corresponding author(s): Amita Sehgal

Last updated by author(s): Nov 27, 2023

Reporting Summary

Nature Portfolio wishes to improve the reproducibility of the work that we publish. This form provides structure for consistency and transparency in reporting. For further information on Nature Portfolio policies, see our Editorial Policies and the Editorial Policy Checklist.

Statistics

For all statistical analyses, confirm that the following items are present in the figure legend, table legend, main text, or Methods section.

n/a	Confirmed	
☐	☒	The exact sample size (*n*) for each experimental group/condition, given as a discrete number and unit of measurement
☐	☒	A statement on whether measurements were taken from distinct samples or whether the same sample was measured repeatedly
☐	☒	The statistical test(s) used AND whether they are one- or two-sided<br>*Only common tests should be described solely by name; describe more complex techniques in the Methods section.*
☐	☒	A description of all covariates tested
☐	☒	A description of any assumptions or corrections, such as tests of normality and adjustment for multiple comparisons
☐	☒	A full description of the statistical parameters including central tendency (e.g. means) or other basic estimates (e.g. regression coefficient) AND variation (e.g. standard deviation) or associated estimates of uncertainty (e.g. confidence intervals)
☐	☒	For null hypothesis testing, the test statistic (e.g. *F*, *t*, *r*) with confidence intervals, effect sizes, degrees of freedom and *P* value noted<br>*Give P values as exact values whenever suitable.*
☒	☐	For Bayesian analysis, information on the choice of priors and Markov chain Monte Carlo settings
☒	☐	For hierarchical and complex designs, identification of the appropriate level for tests and full reporting of outcomes
☒	☐	Estimates of effect sizes (e.g. Cohen's *d*, Pearson's *r*), indicating how they were calculated

Our web collection on statistics for biologists contains articles on many of the points above.

Software and code

Policy information about availability of computer code

Data collection	Sleep data was collected using a Drosophila Activity Monitoring (DAM) System (TriKinetics, Waltham, MA)
Data analysis	All analysis codes used have been identified/cited and are publicly available. ImageJ/FIJI : http://fiji.sc; Ilastik: https://www.ilastik.org; SCAMP Matlab analysis: https://academics.skidmore.edu/blogs/cvecsey/

For manuscripts utilizing custom algorithms or software that are central to the research but not yet described in published literature, software must be made available to editors and reviewers. We strongly encourage code deposition in a community repository (e.g. GitHub). See the Nature Portfolio guidelines for submitting code & software for further information.

Data

Policy information about availability of data

All manuscripts must include a data availability statement. This statement should provide the following information, where applicable:

- Accession codes, unique identifiers, or web links for publicly available datasets
- A description of any restrictions on data availability
- For clinical datasets or third party data, please ensure that the statement adheres to our policy

The data that support the findings of this study have been included as a part of the supplementary data.

Research involving human participants, their data, or biological material

Policy information about studies with human participants or human data. See also policy information about sex, gender (identity/presentation), and sexual orientation and race, ethnicity and racism.

Reporting on sex and gender	N/A
Reporting on race, ethnicity, or other socially relevant groupings	N/A
Population characteristics	N/A
Recruitment	N/A
Ethics oversight	N/A

Note that full information on the approval of the study protocol must also be provided in the manuscript.

Field-specific reporting

Please select the one below that is the best fit for your research. If you are not sure, read the appropriate sections before making your selection.

☒ Life sciences ☐ Behavioural & social sciences ☐ Ecological, evolutionary & environmental sciences

For a reference copy of the document with all sections, see nature.com/documents/nr-reporting-summary-flat.pdf

Life sciences study design

All studies must disclose on these points even when the disclosure is negative.

Sample size	Sample sizes in each experiment were based on the number of samples that we were able to collect/dissect/process within 2hrs, while including all groups to be statistically compared to one-another. Except for sleep, where representative experiments are shown, all replicates have been pooled and all data is shown.
Data exclusions	For sleep/ activity flies were excluded if they died before the end of the experiment. For imaging, damaged brains or those with weak/absent fluorescence (due to loss of mounting medium from edge of slide in a few experiments) were excluded from analysis.
Replication	One or more replicates have been completed and analyzed for each experiment. All replicates analyzed have been normalized within-experiment and then pooled in figures and statistical tests, with the exception of sleep data. For sleep, replicate experiments showing statistical significance have been completed, but a single representative experiment with associated statistics is shown. This allows for presentation of raw, rather than normalized data and is standard in the field.
Randomization	Animals were assigned to groups based on genotype. Assignment of animals within-genotype to sleep/circadian groups was random. Sample processing order and animal placement in incubators was evenly distributed whenever possible. Most analysis was done with the help of unbiased computer programs.
Blinding	Blinding was not used and was not relevant since all samples of all groups to be compared were always subjected to the same computer analysis programs with the same parameters.

Reporting for specific materials, systems and methods

We require information from authors about some types of materials, experimental systems and methods used in many studies. Here, indicate whether each material, system or method listed is relevant to your study. If you are not sure if a list item applies to your research, read the appropriate section before selecting a response.

Materials & experimental systems

n/a	Involved in the study
☐	☒ Antibodies
☒	☐ Eukaryotic cell lines
☒	☐ Palaeontology and archaeology
☐	☒ Animals and other organisms
☒	☐ Clinical data
☒	☐ Dual use research of concern
☒	☐ Plants

Methods

n/a	Involved in the study
☒	☐ ChIP-seq
☒	☐ Flow cytometry
☒	☐ MRI-based neuroimaging

Antibodies

Antibodies used: rabbit polyclonal anti-GFP (1:1,000, Invitrogen #A-11122), MDA(Sigma ms monoclonal 11E3, 1:50 #SAB5202544)

Validation: MDA is species-independent. 11E3 staining intensity is increased following H2O2 incubation (https://www.thermofisher.com/antibody/product/Malondialdehyde-Antibody-clone-11E3-Monoclonal/MA5-27560) additional references showing colocalization with oxidative stress/lipid peroxidation: https://www.abcam.com/products/primary-antibodies/malondialdehyde-antibody-11e3-ab243066.html). GFP #A-11122 is also species-independent and shows a complete absence of staining in GFP-negative samples: https://www.thermofisher.com/antibody/product/GFP-Antibody-Polyclonal/A-11122

Animals and other research organisms

Policy information about studies involving animals; ARRIVE guidelines recommended for reporting animal research, and Sex and Gender in Research

Laboratory animals: All fly lines used are publicly available and have been listed in the Methods section. 3-7 day-old female flies were used for all experiments (4- 14 days old at data collection) except qPCR where both males and females were used. UAS-Drp1-RNAi #44155 was from the VDRC, UAS-Drp1-RNAi #51483 was from the BDSC.

Wild animals: No wild animals have been used in this study

Reporting on sex: Sex has been indicated in the methods section. 3-7 day-old female flies were used for all experiments (4- 14 days old at data collection) except qPCR where both males and females were used.

Field-collected samples: No field-collected samples were used in this study

Ethics oversight: Studies using Drosophila melanogaster are not subject to institutional ethical approval

Note that full information on the approval of the study protocol must also be provided in the manuscript.
